# In the battle of the disease: a transcriptomic analysis of European foulbrood-diseased larvae of the Western honey bee (*Apis mellifera*)

**DOI:** 10.1186/s12864-022-09075-6

**Published:** 2022-12-19

**Authors:** Oleg Lewkowski, Anja Poehlein, Rolf Daniel, Silvio Erler

**Affiliations:** 1 Molecular Ecology, Institute of Biology, Martin-Luther-University Halle-Wittenberg, 06099 Halle (Saale), Germany; 2 Department of Genomic and Applied Microbiology & Göttingen Genomics Laboratory, Institute of Microbiology and Genetics, Georg-August-University of Göttingen, 37077 Göttingen, Germany; 3 Institute for Bee Protection, Julius Kühn-Institute (JKI) – Federal Research Centre for Cultivated Plants, 38104 Braunschweig, Germany; 4 Zoological Institute, Technische Universität Braunschweig, 38106 Braunschweig, Germany

**Keywords:** Brood disease, EFB, Gene expression, Immunity, *Melissococcus plutonius*, Metabolism, *Paenibacillus alvei*, RNAseq, Secondary infection, Transcriptomics

## Abstract

**Background:**

European foulbrood is a significant bacterial brood disease of *Apis* sp. and can cause severe and devastating damages in beekeeping operations. Nevertheless, the epidemiology of its causative agent *Melissococcus plutonius* has been begun to uncover but the underlying mechanisms of infection and cause of disease still is not well understood. Here, we sought to provide insight into the infection mechanism of EFB employing RNAseq in in vitro reared *Apis mellifera* larvae of two developmental stages to trace transcriptional changes in the course of the disease, including *Paenibacillus alvei* secondary infected individuals.

**Results:**

In consideration of the progressing development of the larva, we show that infected individuals incur a shift in metabolic and structural protein-encoding genes, which are involved in metabolism of crucial compounds including all branches of macronutrient metabolism, transport protein genes and most strikingly chitin and cuticle associated genes. These changes underpin the frequently observed developmental retardation in EFB disease. Further, sets of expressed genes markedly differ in different stages of infection with almost no overlap. In an earlier stage of infection, a group of regulators of the melanization response cascade and complement component-like genes, predominantly C-type lectin genes, are up-regulated while a differential expression of immune effector genes is completely missing. In contrast, late-stage infected larvae up-regulated the expression of antimicrobial peptides, lysozymes and prominent bacteria-binding haemocyte receptor genes compared to controls. While we clearly show a significant effect of infection on expressed genes, these changes may partly result from a shift in expression timing due to developmental alterations of infection. A secondary infection with *P. alvei* elicits a specific response with most of the *M. plutonius* associated differential immune effector gene expression missing and several immune pathway genes even down-regulated.

**Conclusion:**

We conclude that with progressing infection diseased individuals undergo a systemic response with a change of metabolism and their activated immune defence repertoire. Moreover, larvae are capable of adjusting their response to a secondary invasion in late stage infections.

**Supplementary Information:**

The online version contains supplementary material available at 10.1186/s12864-022-09075-6.

## Background

Uncovering disease mechanisms is crucial to get a deeper insight into infection dynamics and ecological impact of diseases on host population development and host-pathogen interactions. Most important, acquired knowledge contributes to a broader understanding of the implications and conditions in epidemiological scenarios, and enables the development of strategies for disease control on different levels of interventions and appropriate measures to counteract or even prevent disease outbreaks. European foulbrood (EFB) is a widely distributed and significant honey bee brood disease but despite more than one century of research there is still insufficient understanding of this disease on molecular level [1, 2]. Recently, large regional outbreaks of EFB have been reported from e.g. England and Wales or Switzerland and many cases across numerous European countries were recorded and confirmed, and continue to persistently re-emerge in affected regions [2]. Other global areas like the United States also observe significant increase of EFB cases (pers. comm. Marla Spivak). Changes in beekeeping practises, agricultural intensification [3] and expanding global trade with worldwide accelerated exchange of stock [4] (and not at least climate change), raise presentiments of an upsurge of new epidemics and endemic outbreaks. This causes a pressing need to shed light on the basic mechanisms, the course and the most significant factors contributing to severe infections and disease.

Besides an urgency of developing efficient programs and treatment methods to prevent and counteract new outbreaks, several features of the Gram-positive bacterium *Melissococcus plutonius,* the causative agent of EFB, make it an interesting and promising model pathogen to illuminate general pathogenic mechanisms and counteracting host immune responses ranging from the basic cellular to a behavioural colony level [5, 6]. Its features are the wide range of virulence diversity of different strains [7, 8] and regional enzootic states [9], its resilience to desiccation, high sugar concentrations, and spore-independent viability for months as well as a brood specialization [2].

*M. plutonius* typically infects honey bee larvae in the first days after hatching via ingestion of contaminated food. Then, the larval susceptibility to infection and development of severe disease decreases with larval age until there is no pathogenic effect in the final larval stage before pupation [1, 2]. This high susceptibility of young larvae is also observed in another severe brood diseases, i.e. American Foulbrood (AFB) [2], caused by *Paenibacillus larvae,* suggesting a similar, host-dependent cause of limited infectivity (or a pathogen barrier) in the late larval stages for EFB and AFB, respectively. Although, different mechanisms of resistance and implicated factors have been discussed in former studies, the most critical factor seems to be the pathogen multiplication time until pupation with a potential clearance of the pathogen and a lag of bacterial toxin release [8, 10]. Former studies reported presumably EFB-resistant (or rather tolerant) larvae found in naturally as well as experimentally infected colonies, an observation we also regularly made in our in vitro studies [6–8, 11–13]. These larvae survive pathogen infestation, succeed to pupate (after defecation) and finally emerge as adult workers. A fraction of these workers is seemingly underweight but some of them are morphologically comparable to non-infected individuals. Infections usually depend not solely on the inoculation doses of the infective agent but on many factors like timing of infection, the genotype of the pathogen, the actual susceptibility of the host represented by its genotype and constitution, and also on stochastic processes [14, 15].

Different hypotheses for the observed defence in larvae have been considered, encompassing any layer of immune defence including e.g. the variation in physical barrier properties of the gut, immune system activity comprising immune effector gene expression differences in larvae [7, 13, 16]. Further, food composition and worker added substances have been brought forward as potential colony level defences conveying individual resistance of the brood [17]. Aside from that, a mechanism was suggested where an infestation of larvae leads to a host-pathogen competition for nutrients [11, 12]. In this scenario, the multiplication of the bacterium inside the food mass leads to potential malnutrition, developmental retardation and ultimately starvation and death of the infested larva like hypothesised and substantiated by histological examinations [18–20]. Starvation of infected larvae also indicates early stages of *P. larvae* infections. This was implied by a proteomic study in which multiplying vegetative cells of *P. larvae* metabolize glucose and fructose, the main carbohydrates of food jelly, without detectable tissue damage [21]. Although, *M. plutonius* seems to adhere to the peritrophic matrix (PM) it is clearly contained inside the gut lumen and does not breach the gut epithelium barrier until the larva dies and decay starts [20, 22]. This characteristic course is distinctly different from the disease mechanism of AFB. In AFB the causative agent *P. larvae* actively attacks and breaches the PM structure and the ventricular epithelium, and ultimately invades the haemocoel of the infected larvae [23]. This elicits the typical immune response observed in AFB affected larvae while a response in *M. plutonius* infection was not demonstrated so far [5, 16].

Here, we present an experimental in vitro approach to get a general overview of the expressional response of honey bee larvae artificially infected with *M. plutonius* and a common secondary invader, *Paenibacillus alvei*. This enables an onset of a deeper understanding of the EFB disease infection mechanism and the immune response on a physiological level.

## Results

Bacterial infections may elicit complex and profound systemic responses with far reaching consequences for the attacked organisms. In honey bee larvae, infection with a pathogenic strain of *M. plutonius* often leads to a retardation of growth and therefore interfering with larval metabolism and development with a fatal outcome in severe infections [1].

**Fig. 1:**
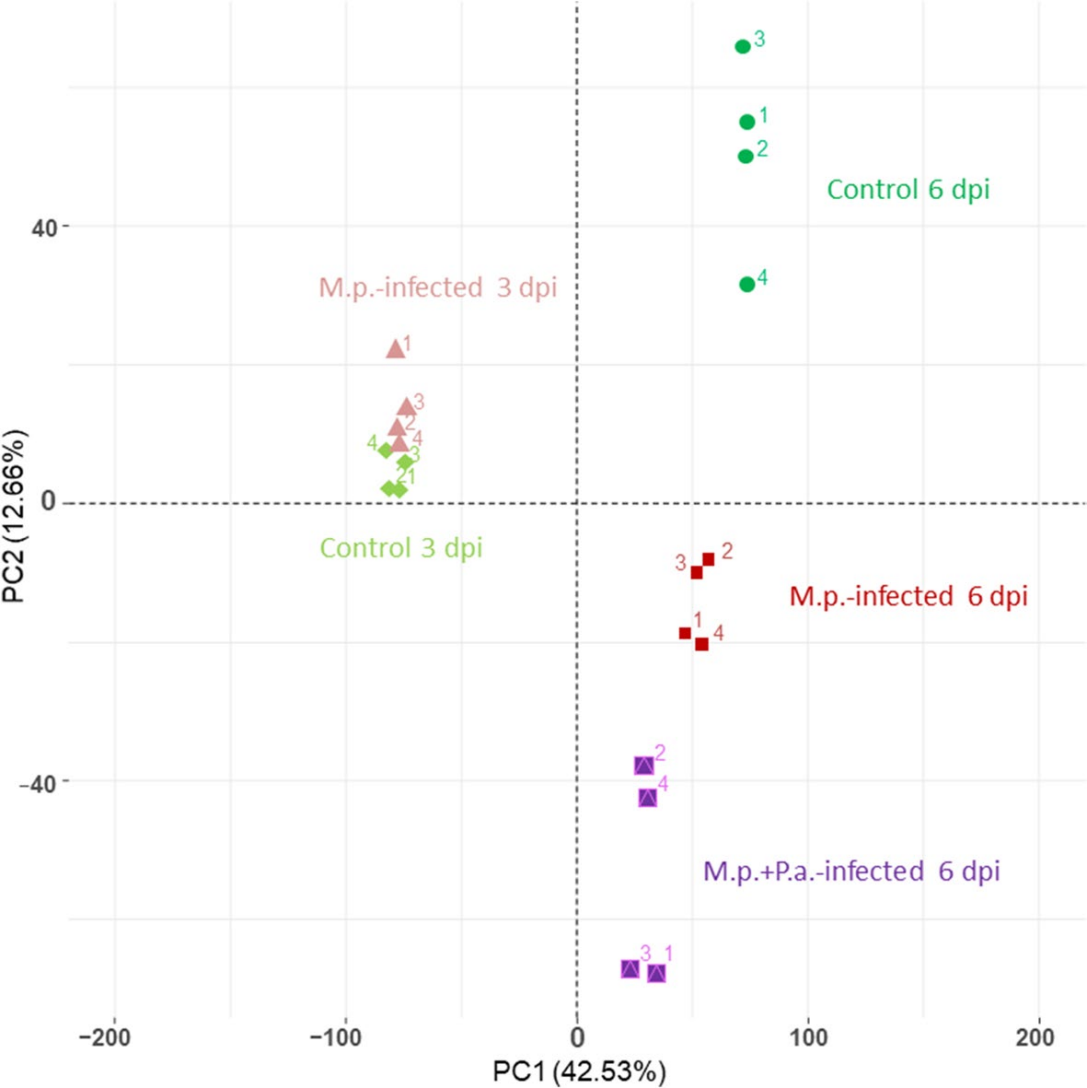
PCA with filtered data set (batch effects removed by RUVr method from the RUVSeq R package). Data sets clearly cluster in respective groups and are markedly separated in larval age groups (3 dpi vs. 6 dpi), while a strong pronounced separation of infected and control samples without an overlap was only observed for 6 dpi. Numbers refer to replicate of group. For more details see Table S 1 (Additional file 3) and Fig. S 8 (Additional file 1) where colors correspond to the same groups, respectively. M.p. – *M. plutonius*, P.a. – *P. alvei*

**Fig. 2:**
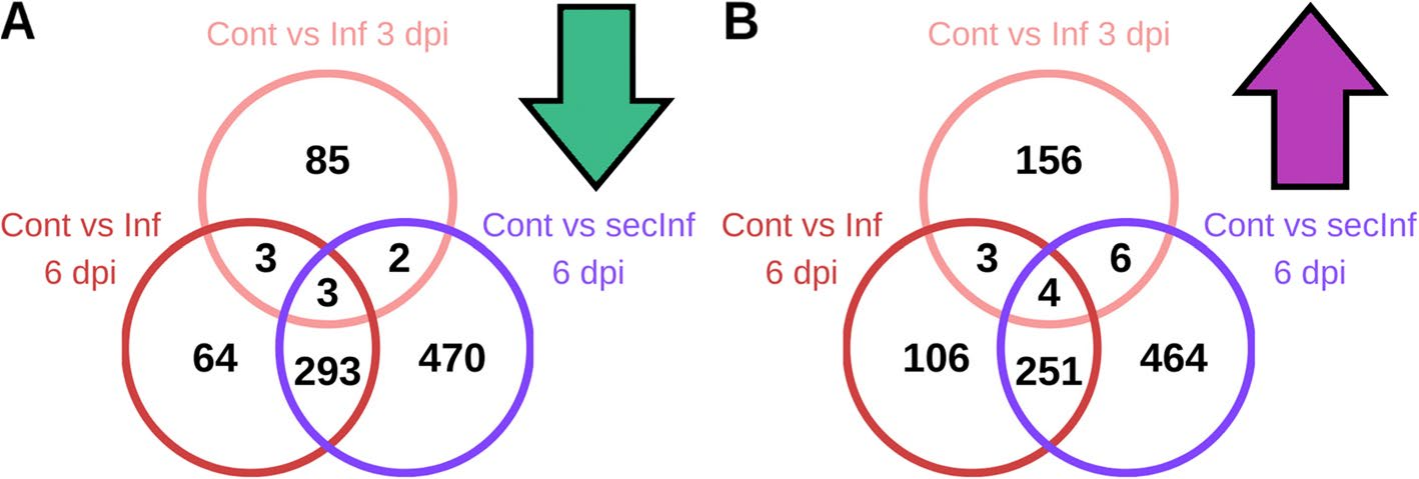
Overlap of single group contrasts of control and infected larvae. **a** Down-regulated DEGs and **b** Up-regulated DEGs. Expressional differences with a high fold change (FC > > 2). (Cont: control, Inf: *M. plutonius*-infected, secInf: *M. plutonius* infected + *P.alvei*)

A principal component analysis (PCA) revealed that all larvae samples from the same treatment groups clustered together after batch effect correction. The data set was strongly separated by larval age (day 4 = 3 days post infection (dpi) vs. day 7 = 6 dpi, 42.53% of total variance) in one dimension, rather than by infection (12.66% of total variance) in the young larvae, compared to the second sampling date where older larvae were clearly separated in distinct clusters (Fig. 1). These results illustrate that expressional developmental changes over time elicit a stronger effect than treatment, i.e. infection. Interestingly, we also find separation of clusters in our correlation of larval weight and *M. plutonius* plasmid copy number (Fig. S 1, Additional file 1). Moreover, comparing DEG sets of contrasts of day 3 (Cont vs. Inf on 3 dpi) and day 6 post infection (Cont vs. Inf; Cont vs. secInf on 6 dpi), a strong difference of expressional patterns was evident with almost no overlap of DEGs between both sampling time points (FDR < 0.05, − 0.585 < log_2_FC > 0.585; Fig. 2).

In a functional annotation clustering (FAC) approach, we further focused on the developmental transition in mRNA abundances in the treatment groups. We first addressed and illustrated the transcriptional change between two time points in the larval development (3 dpi and 6 dpi) and compared the respective differentially expressed gene (DEG) set contrast of 3 dpi vs. 6 dpi of the controls and the contrast of 3 dpi vs. 6 dpi of the *M. plutonius* infected. Then we focused on mRNA abundance of candidate genes of transport/storage proteins and innate immune system genes, which were shown to be a part of a general response in other infections [5, 21]. Finally, we analysed the effect of a secondary infection with *P. alvei*.

### Correlation of weight and expressed genes

Overall, transcriptional activity was increased in lighter and hence younger larvae where mRNA levels of 1110 genes were positively and 2310 negatively significantly correlated with larval weight (Fig. S 2, Additional file 1; Table S 1, Additional file 3). For the positively weight correlated genes, highest ranking terms in the FAC analysis were associated with cellular transport, endocytosis (GO:0035091, GO:0016192, GO:0006886, GO:0035091, ame04144) and lysosome (ame04142) as well as fatty acid degradation (ame00071). In contrast, the negatively weight correlated genes and therefore stronger expressed in young and lighter larvae were mostly involved in splicing, translation and in general RNA turnover associated terms, most strikingly represented by a large set of ribosome protein-encoding genes (ame03010) with consistent correlation coefficients. The other large group of expressed genes is involved in oxidative phosphorylation (ame00190) (for additional details see Table S 1, Additional file 3).

### Functional annotation clustering in temporal expressional shift

**Fig. 3:**
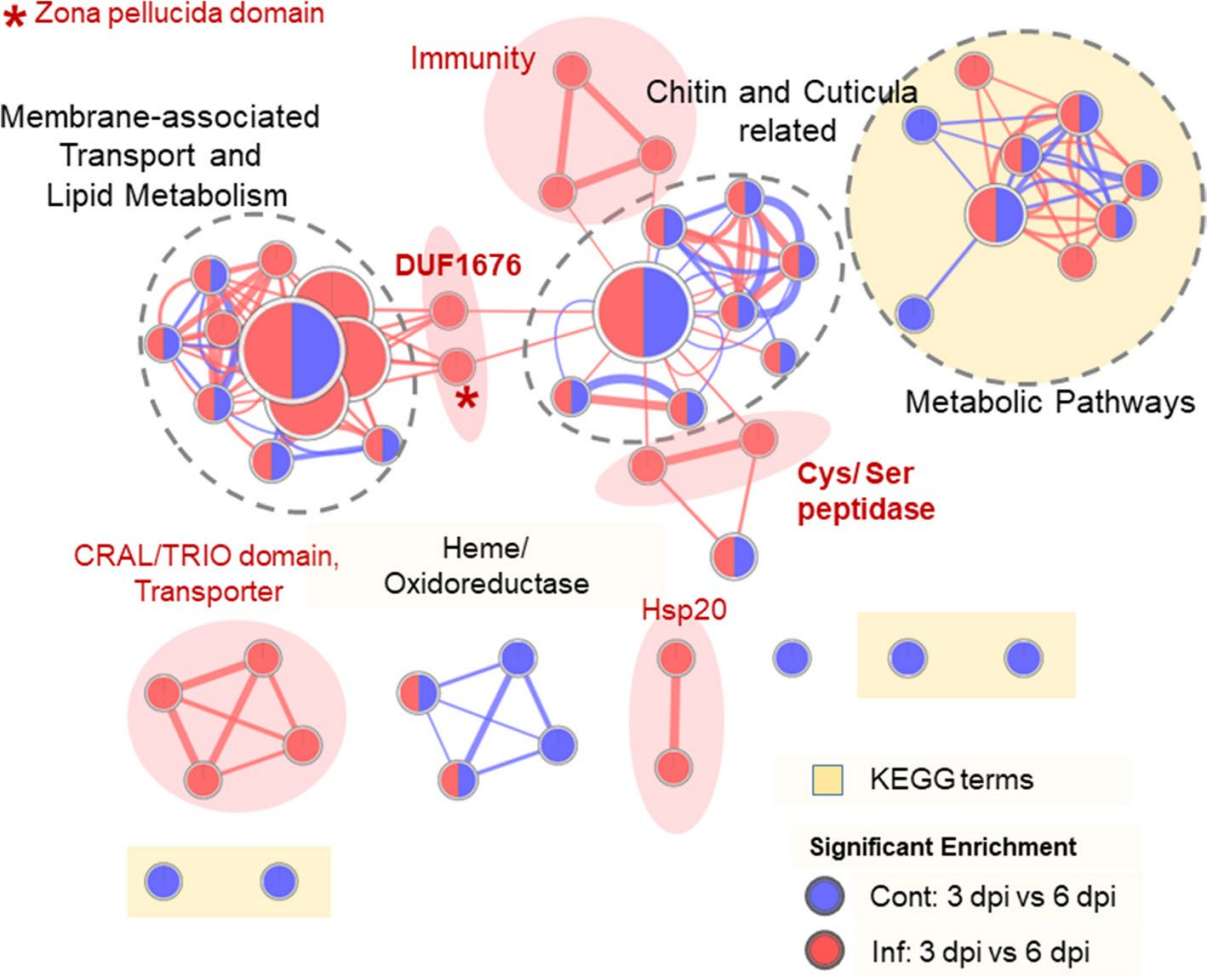
Enrichment network of the DAVID functional annotation analysis. Terms are represented by circles and overlap of terms is represented by node connection resulting in clusters of terms with overlapping functions. Circle size is related to enriched gene set size and edge width is positively associated with overlap of gene sets between the respective terms. For detailed enrichment network see Fig. S 11 (Additional file 2)

As a general overview of transcriptional change in controls and infected larvae, we performed an *Apis mellifera* specific FAC analysis to identify enriched terms and retrieve functional information of enriched genes from the DEG sets (Table S 2, Additional file 4). In total, we identified 3070 DEGs (*p* < 0.05, FDR-corrected: − 1 < log_2_FC > 1) which were significantly regulated. 2090 of the 2255 DEGs in the control group and 1712 of 1848 DEGs in the infected group (Table 1, Fig. S 3, Additional file 1), with *A. mellifera* specific accession in the DAVID database and a differential expression over time (3 dpi vs. 6 dpi, respectively; 1251 DEGs overlapping between both contrasts), were included in our FAC analysis (Table S 2, Additional file 4). FAC yielded 9 annotation clusters containing significant terms (87 in total) for the change of mRNA levels of the controls and 15 annotation clusters (73 in total) for the infected group (*p* < 0.1; Table S 2, Additional file 4). For both controls and infected the two highest-ranking clusters were associated with cuticle proteins, extracellular localisation and chitin binding, illustrating the importance in larval development and the body and gut lining (Table 2; Fig. 3). This was also substantiated by the other categories where the highest-ranking significant terms from Molecular Function (GO:0042302, GO:0008061) and Biological Processes (GO:0006030, GO:0006633) were associated with chitin binding and metabolism. Chitin-binding proteins (CBP) like perithrophins and cuticular proteins but also mucins are a constitutive part of these extracellular structures. In our study, several CBPs undergo significant differential regulation in infected larvae compared to controls (Table S 3, Additional file 5). Corresponding, in the overlap of gene ontology (GO) terms of controls and *M. plutonius* infected the significant terms for “Cellular localisation” were identified as the extracellular region and the membrane (GO:0005576, GO:0016021; Table 2).

**Table 1:** Group contrasts of significantly differentially expressed genes and transcripts

Comparisons	DE genes	DE transcripts
Control 3 dpi vs. Infected 3 dpi	168	100
Control 6 dpi vs. Infected 6 dpi	337	341
Control 3 dpi vs. Control 6 dpi	2255	3012
Infected 3 dpi vs. Infected 6 dpi	1848	2473
Control 6 dpi vs. secondary Infection 6 dpi	601	675
Infected 6 dpi vs. secondary Infection 6 dpi	81	65
3 dpi vs. 6 dpi	1871	3284
Control vs. Infected	101	73

*p* < 0.05, FDR-corrected; Fold change: −1 < log_2_FC > 1; 3 dpi vs 6 dpi = (Cont 3 dpi + Inf 3 dpi)/2 - (Cont 6 dpi + Inf 6 dpi + secInf 6 dpi)/3; Control vs. Infected = (Cont 3 dpi + Cont 6 dpi)/2 - (Inf 3 dpi + Inf 6 dpi)/2; For details on group contrast statistics see Table S 11 (Additional file 13)

**Table 2:** Functional annotation clustering summary of temporal expressional change

GROUP	TOTAL	TERM
Unique Control	7	GO:0005506 ~ iron ion binding
		ame00030:Pentose phosphate pathway
		ame00520:Amino sugar and nucleotide sugar metabolism
		ame00561:Glycerolipid metabolism
		ame00670:One carbon pool by folate
		ame04142:Lysosome
		ame04512:ECM-receptor interaction
Controls + Infected overlap	12	GO:0005576 ~ extracellular region
		GO:0006030 ~ chitin metabolic process
		GO:0006633 ~ fatty acid biosynthetic process
		GO:0008061 ~ chitin binding
		GO:0016021 ~ integral component of membrane
		GO:0020037 ~ heme binding
		GO:0042302 ~ structural constituent of cuticle
		ame00010:Glycolysis / Gluconeogenesis
		ame01100:Metabolic pathways
		ame01130:Biosynthesis of antibiotics
		ame01200:Carbon metabolism
		ame01230:Biosynthesis of amino acids
Unique Infected	4	GO:0005215 ~ transporter activity
		GO:0045087 ~ innate immune response
		ame00260:Glycine, serine and threonine metabolism
		ame00620:Pyruvate metabolism

Control larvae (Cont 3 dpi vs. Cont 6 dpi), *M. plutonius*-infected larvae (Inf 3 dpi vs. Inf 6 dpi) and overlapping significant GO- and KEGG-pathway terms (*p* < 0.1, FDR-corrected). For more details on FAC see Table S 2 (Additional file 4) and Fig. S11 (Additional file 2)

Metabolism related transcriptional changes (3 dpi vs. 6 dpi), particularly represented by Kyoto Encyclopedia of Genes and Genomes (KEGG) terms, significantly enriched in both control and infected groups, involved all major branches like amino acid, carbohydrate and lipid metabolism. Although, most pathways overlapped between controls and infected individuals, there were some terms enriched uniquely and/or expressed exclusively for one or the other group (Table 2). In infected larvae several pathways were differentially regulated on expressional level. In general, in the infected group (3 dpi vs. 6 dpi) just 109 genes (154 in controls) of the parent term “Metabolic pathways” (ame01100) were significantly regulated over time with just 84 genes overlapping in infected and controls and 25 genes showing significant expression in the infected group exclusively (Table 3). This points to a disruption or temporal shift of metabolic pathway regulation in infected larvae. The differentially enriched term “Integral component of membrane” (GO:0016021) is connected to metabolism by the pronounced role of membrane located proteins in lipid metabolism (GO:0006633 = fatty acid biosynthetic process) but also crucial substrate transporter proteins (Table S 2, Additional file 4).

**Table 3:** Significantly enriched KEGG and InterPro terms of DAVID functional annotation in temporal expressional change

			Overlaps		
	Cont	Inf	Cont	Both	Inf
KEGG pathways					
ame00010: Glycolysis / Gluconeogenesis	**20**	**12**	9	11	1
ame00030: Pentose phosphate pathway	**11**	6	6	5	1
ame00260: Glycine, serine and threonine metabolism	8	**10**	2	6	4
ame00520: Amino sugar and nucleotide sugar metabolism	**15**	–	15	–	–
ame00561: Glycerolipid metabolism	**14**	9	5	9	0
ame00620: Pyruvate metabolism	12	**11**	3	9	2
ame00670: One carbon pool by folate	**7**	5	2	5	0
ame01100: Metabolic pathways	**154**	**109**	70	84	25
ame01130: Biosynthesis of antibiotics	**52**	**40**	19	33	7
ame01200: Carbon metabolism	**31**	**21**	15	16	5
ame01230: Biosynthesis of amino acids	**25**	**17**	10	15	2
ame04142: Lysosome	**19**	14	9	10	4
ame04512: ECM-receptor interaction	**9**	–	9	–	–
InterPro					
IPR000618:Insect cuticle protein	**25**	**23**	3	22	1
IPR001071:Cellular retinaldehyde binding/alpha-tocopherol transport	6	**8**	0	6	2
IPR001251:CRAL-TRIO domain	–	**10**	–	–	10
IPR001254:Peptidase S1	11	**13**	2	9	4
IPR001436:Alpha crystallin/Heat shock protein	7	**9**	0	7	2
IPR001507:Zona pellucida domain	–	**8**	–	–	8
IPR002557:Chitin binding domain	**21**	**18**	5	16	2
IPR005828:General substrate transporter	**20**	**16**	8	12	4
IPR006631:Protein of unknown function DM4/12	**9**	**10**	0	9	1
IPR009003:Trypsin-like cysteine/serine peptidase domain	11	**13**	2	9	4
IPR011074:CRAL/TRIO, N-terminal domain	7	**9**	0	7	2
IPR012132:Glucose-methanol-choline oxidoreductase	**11**	8	5	6	2
IPR012464:Protein of unknown function DUF1676	–	**12**	–	–	12
IPR020846:Major facilitator superfamily domain	**47**	**33**	21	26	7

Uniquely assigned (Cont = Cont [3 dpi vs. 6 dpi], Inf = Inf [3 dpi vs. 6 dpi]) and overlapping genes (represented in both contrasts = Both) of temporal expressional change in 3 dpi vs. 6 dpi controls or infected, respectively. Bold numbers represent significant enrichment (*p* < 0.1; FDR-corrected) of the respective terms in functional annotation clustering analysis. Missing terms in the respective contrast are represented by a minus sign. For further details and term associations see Fig. 2, Table S 2 (Additional file 4)

In the course of infection (Inf 3 dpi vs. Inf 6 dpi) significantly expressed gene sets of the carbon metabolism (ame01200) as well as the overlapping pentose phosphate pathway (ame00030) and glycolysis/gluconeogenesis (ame00010) are strongly reduced compared to controls (Cont 3 dpi vs. Cont 6 dpi). In contrast, the expressional pattern of the pyruvate pathway (ame00620) gene set composition seems to be sustained for the most part. All these pathways are crucial in storage and conversion of energy reserves for development and metamorphosis in insect larvae. Moreover, there are two functionally related InterPro terms of transporter proteins with a differential enrichment, the “General substrate transporters” (IPR005828) and the “Major facilitator superfamily” (IPR020846) (Table 3). These terms comprise two overlapping sets of membrane transport proteins of various molecules but most importantly including carbohydrate and other organic molecule transporters. Specifically, the change in the trehalose and glucose transporter expression points to a shift in carbohydrates conversion, storage and distribution in infected larvae over time (for additional details see Table S 2, Additional file 4).

Temporal expression changes of several genes from different classes of protein families (InterPro category) were significantly enriched in the infected larvae particularly Trypsin-like cysteine/serine like peptidase (IPR009003), CRAL/TRIO domain (IPR001251), heat shock protein 20 (Hsp20) domain (IPR002068), DUF1676 (IPR012464) and Zona pellucida (ZP) domain (IPR001507) containing protein genes (Fig. 3, Table 3; for additional details Table S 2, Additional file 4).

### Specific proteins involved in larval development

**Fig. 4:**
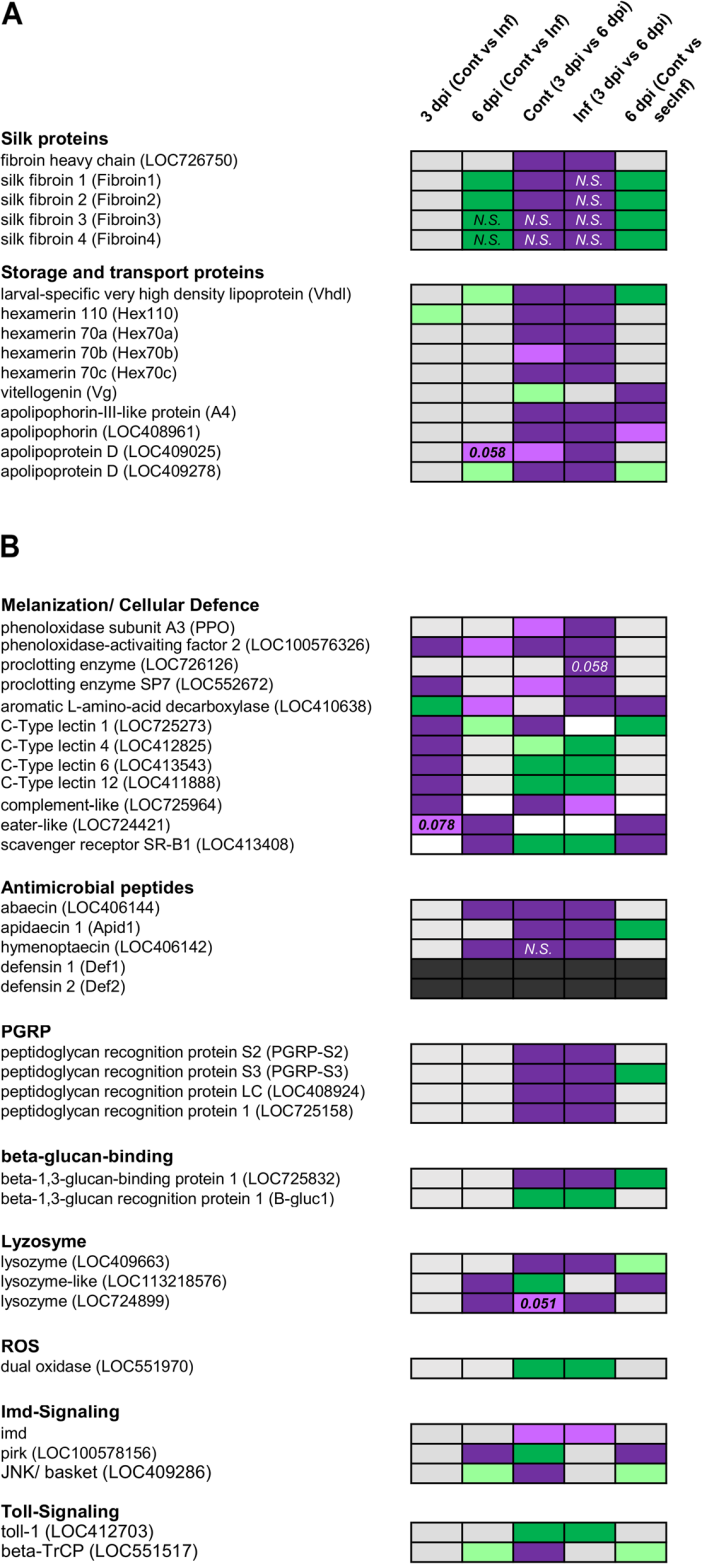
Candidate gene expression of **a** larval specific and storage/transport protein genes and **b** canonical immune effector and signalling genes in single group comparisons. (Dark green: down-regulation, FC > 1; light green: down-regulation, FC < 1; purple: up-regulation, FC > 1; pink: up-regulation, FC < 1; light grey: not differentially regulated; dark grey: not detected). In case of a borderline significance the *p*-value is shown for the respective comparison. Expressional differences with a high fold change (FC > > 2) but not significant values are marked with N.S. For more details see Table S 6 (Additional file 8)

Insect larvae possess specific storage and transport proteins. In honey bees, proteins like hexamerines and a Very high density lipoprotein (VHDL) are strongly expressed in larvae and are accumulated in the haemolymph and the fat body preceding metamorphosis, respectively [24, 25]. Other functional proteins, like fibroins are massively synthesised in late-stage larvae prior to the spinning stage and are indicative for proper developmental progress [26]. We found a significant expressional change of the corresponding protein-encoding genes when comparing young 3 dpi and old 6 dpi larvae in both, infected and control groups, respectively (Fig. 4A). While expression of genes encoding storage and transport proteins was mostly unchanged in infection with *M. plutonius* as well as in secondary infected larvae (*M. plutonius* + *P. alvei* treatment), *silk fibroin* mRNA levels were massively down-regulated with a factor of 24 to more than 40 in infected larvae. Notably, secondary infection (*M. plutonius* + *P. alvei*) led to a similar reduced *silk fibroin* expression in old larvae while storage protein mRNA stayed unchanged and even dropped for *vhdl* and therefore was failed to be regulated (Fig. 4A).

In our FAC analysis, we found several genes encoding groups of proteins, which were solely enriched in temporal expressional change of infected larvae (Table 4). DUF1676 is characteristic for the *osiris* gene family which were found to be coincidentally expressed with cuticle deposition in development but are also suspected to be involved in other functions like immunity [27]. We found a number of *osiris* genes to be significantly regulated over time in the control group (3 dpi vs 6 dpi) and even more pronounced in the infected larvae of up to a 1000-fold change. While at 6 dpi there were no differences between control and infected group, the 3 dpi contrast showed several *osiris* genes to be significantly up-regulated in the infected individuals (Table S 4, Additional file 6). The Zona pellucida (ZP) domain is associated with diverse functions but primarily is present in membrane-bound proteins with filaments and/or matrix related context [28]. In infected larvae, genes of ZP domain containing proteins were mostly down-regulated over time compared to the control group. All genes belonging to the stress responsive group of Hsp20 chaperones, including several *l(2)efl* genes, were up-regulated in older infected individuals (Table 4).

**Table 4:** Differentially enriched InterPro terms of functional groups from FAC analysis of significant DEGs

		Contrast	
Enriched protein group (associated and overlapping terms)	Regulation	Cont 3 dpi vs 6 dpi	Inf 3 dpi vs 6 dpi
IPR001251: CRAL-TRIO domain (IPR001071:Cellular retinaldehyde binding/alpha-tocopherol transport, IPR011074:CRAL/TRIO, N-terminal domain)	up	2	**3**
	down	5	**6**
	no	0	0
IPR001254: Peptidase S1 (IPR009003:Trypsin-like cysteine/serine peptidase domain)	up	6	6
	down	4	**7**
	no	3	**0**
IPR001436: Alpha crystallin/Heat shock protein	up	6	**9**
	down	1	**0**
	no	2	**0**
IPR001507: Zona pellucida domain	up	1	1
	down	4	**7**
	no	3	**0**
IPR012464: Protein of unknown function DUF1676	up	0	0
	down	11	**12**
	no	1	**0**

DEGs in groups may overlap. Differential regulation in temporal change of infected individuals is highlighted in bold. For more details on FAC see Table S 2 (Additional file 4)

### Innate immune response and immune pathway signalling

#### Toll and Imd/JNK signalling

Honey bee larvae express a strong immune response upon a challenge with pathogens from different types of organisms [5]. In our experimental set up, we therefore expected an activation of the Toll and Imd pathways, which are responsive to peptidoglycan containing Gram-negative and Gram-positive bacteria, like *M. plutonius.* In regard of the mRNA abundance of Toll and Imd signalling pathway related genes of the control groups (Cont 3 dpi vs. Cont 6 dpi), we observed a strong up-regulation of bacteria specific pattern recognition receptors (PRR) for both Toll and Imd pathway over time while a fungi specific PRR was significantly down-regulated (*b-gluc1,* FC = 2.71; Fig. 4B). Further, a decrease of the Imd inhibitor *pirk* and the *dual oxidase* expression was observed (Fig. 4B). While *pirk* is significantly down-regulated in control larvae over time (FC = 4.0, *p* = 0.0053), a temporal change in the infected groups was not recorded. (Inf 3 dpi vs. Inf 6 dpi; FC = 0.01, *p* = 0.99; Table S 5, Additional file 7).

#### Innate immunity

We detected only two differentially expressed canonical immunity genes in samples of day 3 post infection, *phenol oxidase-activating factor 2* and *proclotting enzyme SP7*, serine proteases that were shown to be involved in prophenoloxidase (PPO) activation [29]. In addition, several complement component associated protein genes like C-type lectins (CTL1, CTL4, CTL6, CTL12) and another complement-like protein (LOC725964) were two- to four-fold up-regulated in infected individuals (Fig. 4B; Table S 6, Additional file 8).

In our experimental set up the onset of immune effector expression was observed with progressing infection in the 6 dpi larvae. We detected increased mRNA levels of several antimicrobial peptides (AMPs) like *hymenoptaecin* (7-fold) and *abaecin* (more than 2-fold) and *lysozymes* (LOC113218576, LOC724899; 3.5-fold and 2.5-fold) in older infected larvae (Fig. 4B). Defensins comprise a prominent and widely conserved AMP family and usually are expected to be expressed upon an immune challenge. In our data set *defensin 1* and *2* where not represented or just inconsistently and in negligible read count numbers. The expressional differences of regulated AMPs (including *apidaecin*) in treatment groups are exceeded by a magnitude when compared to the differential expression of these AMPs over time (controls as well as infected). Here, in both controls and infected we found a strong increase of mRNA levels up to more than 6000 times (*abaecin*) higher than in 3 dpi larvae.

However, the most striking transcriptional change in 6 dpi infected larvae (both just *M. plutonius* and secondarily infected) was an increase in expression of cellular defence associated genes *eater-like* (LOC724421) and a *scavenger receptor class B member 1* (LOC413408; also *peste* homologue) compared to 6 dpi controls (Fig. 4B). Particularly, *eater-like* is neither regulated in the controls nor infected larvae over time (3 vs 6 dpi) and therefore seems to be a specific expressional response to the induced infection.

### Feeding behaviour and hormone-associated genes

In *M. plutonius* infections we usually observe diseased larvae with a strong reduction in body weight and cessation of feeding [6, 7]. Hence, we were interested in the expression of candidate genes related to feeding behaviour. Comparing 6 dpi controls and 6 dpi infected larvae we found differences in mRNA levels of genes, which previously have been associated with feeding behaviour and nutritional stress [30, 31]. Particularly, neuropeptide F (*npf*) was significantly higher expressed in 6 dpi infected larvae (3.2 times, FDR = 0.023) and even more pronounced in larvae secondary infected with *P. alvei* (5.3 times, FDR < 0.001) compared to controls. These results are substantiated by a differential expression of the serine protease *scarface* (*scaf*) gene, which was included in the group of IPR001254: Peptidase S1 term of our FAC analysis. *Scaf* was down-regulated in the 6 dpi *M. plutonius* infected group by a factor of 1.8 compared to 6 dpi controls (FDR < 0.001). In *D. melanogaster Scaf* acts as insulin signalling independent neuromodulator in sugar deprived states and is expressed in *scaf-*specific specialized neurons [31]. These data are further supported by our FAC of a transcriptional change in significantly enriched genes categorised in GO terms related to metabolism and transport of carbohydrates (Table S 2, Additional file 4). Interestingly, 6 dpi *M. plutonius*-infected larvae showed an altered expression of genes involved in hormone synthesis pathways (Table S 7, Additional file 9; Fig. S 4, Additional file 1). One of the important groups here are heme-containing enzyme genes. Several of these genes, which often possess oxidoreductase activity and may also be involved in detoxification of xenobiotics, were differentially expressed between infected and controls over time (3 dpi vs 6 dpi; Table S 8, Additional file 10). Specifically, altered expression of two *farnesol dehydrogenases* genes (LOC412458, LOC725489), enzymes involved in the juvenile hormone (JH) synthesis pathway, mediating the oxidation of farnesol to farnesal [32], may have implications for timing of larval development, successful metamorphosis and ultimately survival. Another important regulator of larval development is 20-Hydroxyecdysone (20-HE). A perturbation of the synthesis of active 20-HE through inhibition of the hydroxylation of ecdysone is indicated by down-regulated cytochrome P450 314A1 (also called *shade*) gene expression by a factor of two (Table S 7, Additional file 9; Fig. S 4, Additional file 1).

### Secondary infection

We compared transcriptional levels of the DEG sets of solely *M. plutonius* infected 6 dpi (Cont vs Inf) with *M. plutoinus* and secondarily *P. alvei* infected (Cont vs secInf) 6 dpi larvae to determine the effect of secondary infection on gene expression patterns. We did not find significantly increased mortality in secondarily infected larvae in the recent study (compare [7, 8]) but, nevertheless, a marked difference of expressed genes of Cont vs Inf compared to Cont vs secInf individuals. Most DEGs from the Cont vs Inf contrast are also represented by comparable fold changes in the Cont vs secInf contrast, confirming the consistency of the DEG pattern of *M. plutonius* infection (Figs. 2 and 4). FAC analysis with a less restrictive DEG threshold (− 0.585 < log2FC > 0.585; *p* < 0.05; separated up- and down-regulated genes; for additional details see Table S 12, Additional file 14) yielded significant enrichment of 6 terms in the GO and KEGG categories for up-regulated DEGs in Cont vs Inf (348 DEGs) compared to 13 terms in Con vs secInf (696 DEGs). In secondarily infected individuals, most of these terms are functionally associated with metabolism, specifically a large set of KEGG terms related to carbohydrate metabolism and other terms with a function in release of stored energy like the pyruvate metabolism or the citric acid cycle. In contrast, in the solely *M. plutonius* infected group, the set of up-regulated terms is limited to galactose and glucose metabolism. Here, we found rather a more expanded set of down-regulated significant terms with function in transport, chitin binding and oxidoreductase activity (GO:0005215, GO:0008061, GO:0016614). As mentioned above, the response was for the most part comparable in the secondarily infected group but several DEGs were regulated differently (Fig. 4; Table S 6, Additional file 8). The lipoprotein genes of vitellogenin (*vg*) and apolipophorin-III-like protein (*A4*), and to a lesser amount the apolipophorin gene *LOC408961,* were significantly up-regulated in secInf compared to controls (fold change: *vg* = 3.4, *A4* = 3.2, *LOC408961 =* 1.6). Surprisingly, in secondarily infected larvae mRNA levels of one lysozyme gene (*LOC724899*), *abaecin* and *hymenoptaecin* were not different from controls. Furthermore, there was a remarkable down-regulation of *apidaecin* (fold change: 7.1) in the secondarily infected group as well as a decreased expression level of two PRRs (fold change: *PGRP-S3* = 2.0, *LOC725832* = 2.8).

### Regulatory non-coding RNAs

Non-coding RNAs (ncRNA) are involved in gene regulation in a variety of organisms and different types of regulatory RNAs were described in the last couple of decades [33]. Besides many other crucial functions, ncRNAs were shown to play an important role in innate immunity and are involved in pathogenic mechanisms of different pathogens [34]. While only one microRNA Mir9865 was significantly regulated in one group contrast, namely 3 dpi infected vs 6 dpi infected (*p* = 0.015, FC = 2.18, down-regulated), we found that regulation of several putative ncRNAs was strongly dependent on *M. plutonius* infection. In our expressed transcript set, we identified 1399 putative ncRNA specimen in both controls and infected larvae without being exclusive for one group. In total, we identified 8 ncRNAs which were significantly different in their in-group temporal change of abundance in controls versus infected (Table 5).

**Table 5:** Significant expressional change of non-coding RNAs putatively regulated in the course of *M. plutonius*-infection

Target	Adj. *p*-value	Log2 fold change
XR 003306022.1	0.041	1.71
XR 407617.3	0.010	1.60
XR 003304595.1	0.017	1.32
XR 003305626.1	0.003	1.24
XR 120007.4	0.006	1.11
XR 411275.3	0.043	1.01
XR 001703732.2	0.037	−1.13
XR 003305118.1	0.031	−7.24

## Discussion

We provide a transcriptome study on *M. plutonius* and *P. alvei* secondarily infected larvae of the Western honey bee *Apis mellifera* resembling European foulbrood disease with new insights on course of infection and its impact on larval development. First, we confirm previous findings that young larvae are highly active in nutrient conversion and protein synthesis with a high rate of oxidative phosphorylation, gene expression and translation [35]. Heavily enriched sets of genes involved in RNA-turnover and transport further confirm these results (Table S 1, Additional file 3). In older individuals, conversion and allocation of synthesised and stored reserves, including fatty acid degradation, are predominantly marked by processes related to inter- and intracellular trafficking, reflecting the high activity of the fat body cells and preparation to metamorphosis. In the next step of our analysis, we compared the temporal change of functional enriched gene sets of infected and control larvae employing functional annotation clustering.

Most notably, functional annotation clustering (FAC) analysis yielded significant enrichment of chitin metabolism and chitin biosynthesis-associated terms, ranking highest in the categories “Molecular Function” and “Biological Process”. These clusters contain a large group of peritrophines and cuticular protein genes as well as genes of other chitin binding proteins. Chitin turnover occupies a predominant role in larval development as chitin containing structures are repeatedly built up, broken down and recycled to enable larval growth and to constitute the body surface as well as the peritrophic matrix, an important component of the larval ventriculus [36]. Other structural proteins like collagens and laminins are important components of extracellular matrices (ECM), e.g. the lining of organs, and are involved in ECM receptor signalling and cell-to-cell communication [37]. Hence, perturbation of chitin and extracellular structure synthesis and metabolism will most likely affect the development of the larva.

A differential set of genes involved in several branches of metabolism indicate a change and shift in metabolic activity. Gene sets of several pathways presumably failed to be regulated in the same manner in the development (i.e. from 3 dpi to 6 dpi) of infected larvae as compared to control larvae. Here, carbon metabolism-associated pathways, which are involved in the metabolism of fat and glycogen seem to be strongly affected. This is also reflected in a change of associated membrane transport proteins expression. Larvae build up lipid and glycogen stores in fat body cells during the feeding stages and these are increasingly found in the larval trophocytes of the fat body [38]. In general, these resources are accumulated for energy supply and synthesis of structural and metabolic components, which are crucial in metamorphosis. An intruding pathogen elicits an immune system activation with an allocation of these resources [39]. Mounting an immune response is usually predicted to be costly and to trade off with other fundamental functions most strikingly seen under stress conditions like starvation and poor nutrient supply [15]. Whether the developmental retardation in EFB disease is a consequence of allocation of resources to the immune system or a direct result of competition for nutrients with the pathogen needs further investigations.

Two other groups of genes were represented in the FAC analysis and are associated with ECM, DUF1767 domain containing protein-encoding genes (predominantly Osiris family) and Zona pellucida (ZP) domain family genes. Although, their functions are not well understood, these results overlap with transcriptome studies of infections with another honey bee brood pathogen, namely *P. larvae* [40]*.* The most pronounced similarity is the enrichment and differential expression of Chitin-binding Protein (CBP) as well as Osiris family and ZP domain protein genes. This is not surprising as these infections are both located in the gut of the larva and most likely interfere with the larval development as already suspected by Cornman et al. [40]. Hence, this expressional pattern may be a part of a more general mechanism in infections of honey bee larvae. A shift or disturbance in larval development may also be derived from the observed lowered mRNA levels of the fibroins in *M. plutonius* infected and *P. alvei* secondary infected larvae in our study. Silk fibroin is synthesised prior to the spinning stage and used in the beginning of pupation [26]. Beyond that, the observed enrichment of a set of Hsp20 genes may be an indicator of a stress response in infected individuals in the course of the infection. Hsp20 proteins are a family of chaperones, which play a significant role in different physiological processes including the maintenance of cellular proteins under a variety of stress conditions [41].

### Toll and Imd/JNK signalling

The observed decrease of Imd inhibitor *pirk* and the *dual oxidase* expression in untreated larvae is most likely attributable to an immune system activation in preparation to metamorphosis, in which *pirk* transcription decreases and therefore gives rise to immune effector expression like e.g. observed for the elevation of several AMP mRNA levels in the same group contrast. Coincidently, *dual oxidase* is presumably down-regulated to avoid host tissue damage by free reactive oxygen species during tissue restructuring. In contrast, the high *pirk* mRNA levels of infected compared to controls led us suspect an Imd signalling pathway inhibition [42]. Toll and Imd pathways were shown to be selectively activated depending on the composition of the pathogen-associated molecular patterns (PAMPs) with Toll pathway directed against Gram-positive bacteria and fungi and Imd pathway against Gram-negative bacterial cells [5]. Although, this mechanism of strict specificity was challenged, it may explain a reduced activity of the Imd pathway in a late response whereas Toll pathway, potentially selectively recognising the Gram-positive *M. plutonius*, seems to be sustained. Furthermore, activation of these pathways may differ in timing or be sequential [43, 44]. In other insects like *Drosophila melanogaster*, the Toll and Imd pathways are both known to be involved in the activation of cellular defence and melanisation response or at least overlap in regulation [44]. Therefore, this may also hold true for the honey bee, where we find a regulation of genes associated exactly with these defences.

### Immune effector expression

Contrary to our expectations, we did not detect a significant differential immune response, specifically AMPs, in young infected larvae (3 days post infection = 72 h). In previous experiments, we showed that in 1 day-old and 3 days-old larvae infected per os with *M. plutonius*, an expressional change for a set of canonical immune genes of signal or effector proteins was not detectable [16]. Here, our transcriptomic analysis confirms these previous findings, although in future examinations of transcriptional AMP profiles a higher resolution of expressional timing would be desirable, particularly close to the infection time point. In comparison, *P. larvae* infections, causing AFB, the most dreaded bacterial disease of honey bee brood, elicit different immune effectors like AMPs 72 h after inoculation with the infecting agent [5, 40]. Although, we did not detect a significant change of *ppo* expression in infected larvae two putatively PPO cleaving serine proteases were up-regulated in 3 dpi *M. plutonius* infected larvae, presumably increasing the potential for active phenoloxidase (PO) in infection. A proteomic study on AFB diseased larvae demonstrated that in general, PO activity is highest on day 4 post-hatching (corresponding to 3 dpi in our study) and PPO seems to be accumulated in the haemolymph [21]. Although, PO is a well-established immune effector there may be also other functions as well as different pathways of activation. Moreover, several complement component associated protein genes were significantly altered in mRNA abundance with infection, including several C-type lectins (CTL1, CTL4, CTL6, CTL12) and another complement-like protein (LOC725964) in 3 dpi larvae, which have a potential role in opsonization of pathogens and possibly contributing to a PPO activation, a melanization/nodulation reaction and haemocyte-associated cellular defence [45].

On 6 dpi several immune effector genes were significantly up-regulated in infected individuals compared to controls, particularly *hymenoptaecin* and *abaecin* as well as two *lysozymes*. We did not detect *defensin* expressional regulation (Fig. 4B). Although, *defensins* usually are strongly up-regulated in adult bees upon infections [46] it seems to be less responsive in larvae. One reason may be the defensin content of the larval food, which is produced and secreted by the feeding workers [47]. For *Drosophila melanogaster* it was shown that both Toll and Imd pathways are necessary to up-regulate defensin [48]. In our set up, the Imd pathway seems to be inhibited and therefore prohibiting *defensin* expression is a mechanistic explanation for our observation. Moreover, defensins may also be expressed locally [49]. Hence, mRNA level variation in different tissues may remain undetected if measured in whole larvae like in our study.

By comparing the effect of time and treatment on immune effector mRNA levels, the time factor, and hence larval maturation, has by far a stronger effect on expression in both controls and infected individuals. This expressional regulation of immune effectors and other immune pathway genes is most likely due to the preparation for metamorphosis of the late-stage larvae like observed for other holometabolous insects [50]. This rational can also lead to an alternative interpretation of the observed gene expression differences. Hence, difference in immune effector abundance, particularly of AMPs, may also be attributed to a developmental retardation and a shift of developmental phases rather than a strong induced response to infection. This is supported by the expressional profiles of other gene groups like mentioned above. Unfortunately, we are not able to assess the strength of individual factors in this study.

However, the most striking transcriptional change in older infected larvae (both just *M. plutonius* and secondarily infected) was an increase in expression of cellular defence associated genes *eater-like* (LOC724421) and a *scavenger receptor class B member 1* (LOC413408; also *peste* homologue) compared to controls. Eater is a haemocytes-specific Nimrod family cell surface receptor, in particular expressed by the plasmatocyte lineage in *Drosophila melanogaster* [51]*,* a cell type analogous to honey bee granulocytes. Richardson et al. [52] reported that granulocytes are the dominant haemocyte type in honey bee larvae, so elevated expression of *eater* points to an increase in phagocytosis-associated cellular defence against Gram-positive bacteria. Moreover, Eater is the most crucial receptor in phagocytosis of *E. faecalis,* an Enterococcus species closely related to *M. plutonius*. The *scavenger receptor class B member 1* is another protein gene that was shown to be involved in cell surface recognition and phagocytosis of pathogens [53].

Melanization cascade, complement-like and haemocyte-specific receptor gene expression as well as inhibition of the Imd pathway signalling genes integrate well in a mechanism with an activation of a cellular defence involved response in the larval haemolymph and possibly the gut. In *Drosophila* it was proposed that gut tissue residing haemocytes, which are spanning the gut epithelium, may be able to signal a gut infestation to the haemocoel and elicit a systemic response [48]. The honey bee larva is usually not able to stop multiplication of *M. plutonius* in the gut, but it still may be well prepared for a dissemination of bacterial cells into the haemolymph. Although, *M. plutonius* infections are most likely restricted to the larval gut and a spread of *M. plutonius* cells into the haemocoel was not shown so far, this phenomenon was already studied for other bacterial diseases like AFB [23]. An active breaching of the gut epithelium like demonstrated for *P. larvae* is not reported for *M. plutonius,* but a possible leakage of bacterial cells through loss of barrier function in late-stage infections could also be a plausible mechanism for EFB disease progression. Previously, loss of intestine barrier function was connected to enhanced immune system activation, inflammation, aging and reduced survival in *Drosophila* [54, 55]. While younger honey bee larvae seem to rely rather on PPO activation and opsonization of intruders like stated above, older larvae usually have higher numbers of haemocytes and therefore most likely invest in a stronger cellular defence [52]. This may contribute to an explanation why larvae survive better when infected in a later stage of development besides the reduced available time for multiplication of the pathogen inside the gut.

### Behavioural change and hormone associated genes

Infections can have a significant effect on the behaviour of the host. Systemic changes during infection like shifts in metabolism and feeding behaviour are regulated by a network of hormones and neuropeptides, which usually also play a fundamental role in developmental control [56]. We found several indicators of expressional regulation of genes, which correlate with starvation and behavioural change. Several peptides, which are produced in specialized cells in the brain and peripheral tissues, are known to regulate hormone release and elicit behavioural change in insects [56]. *Neuropeptide F* was higher expressed in older infected larvae in both just primarily and secondarily infected larvae compared to controls while the serine protease gene *scarface* was down-regulated in these groups. This expressional pattern is usually associated with modification of feeding including a prolonged larval food uptake before metamorphosis, modulation of larval food foraging behaviour and may delay pupation like shown for other insects (e.g. *Drosophila melanogaster*) [30, 31]. Development and survival of larvae are also affected by the alteration of the synthesis of two hormones, JH and 20-HE, which play a crucial role in larval development and metamorphosis. Hence, differential expression of farnesol dehydrogenases genes as well as the down-regulated cytochrome P450 314A1 (also called *shade*) gene expression may point to an inhibition of the hydroxylation of ecdysone to active 20-HE and an altered JH synthesis. These steps of synthesis and modification are critical prior to the secretion and activity of these hormones. In our study, this is another indication of delay and an explanation for the regularly observed failure of developmental in EFB diseased larvae. An altered juvenile hormone as well as ecdysone synthesis gene expression are in concordance with this interpretation and underpin this view.

### Secondary infection

Besides the primary infection with *M. plutonius*, in EFB several secondary invaders, i.e. opportunistic bacteria joining the causative agent in a later stage of infection, have been identified [2]. Previous studies obtained ambiguous results concerning an effect of the secondary invader, *Paenibacillus alvei*, on larval survival. Some approaches did not find increased mortality in in vitro infection experiments [7, 8] while Giersch et al. [57] could show an effect. Aside from an impact of secondary invaders on host survival there is a potential for an expressional response in secondary infection that does not directly translate to an apparently observable phenotype. In the present study, we did not find significantly increased mortality but a marked difference of expressed genes in secondary infection compared to only primary infected larvae, although a large proportion of DEGs overlapped. Interestingly, while most candidate genes are regulated in the same fashion in both contrasts, the secondary infection induces no significant effect or even a down-regulation of AMPs, some *lysozymes* and PRRs. Further, in contrast to solely *M. plutonuis* infected larvae lipoprotein genes like *vg* and *A4*, which are expected to have a function in immunity, were up-regulated. A secondary infection with *P. alvei* is apparently triggering a much stronger effect on metabolism by transcriptionally up-regulating a broad spectrum of different metabolic pathways compared to solely *M. plutonius* infected individuals. This response is potentially a specific tuning of the immune system and metabolism to a second invading bacterium in infected larvae.

Most noteworthy, significant enrichment of down-regulated hippo signalling pathway genes is indicating an interference of secondary infection with growth and development [58]. Unfortunately, in reference to several not or down-regulated canonical immune genes we cannot differentiate between a specific adjusted larval immune response to a secondary infection with a specific bacterium or a possible immune system inhibition by the secondary invader. This question has to be examined in additional experimental work.

## Conclusion

The results of our analysis point to a change of the metabolic state and a dysregulation or temporal shift of structural and functional protein expression in EFB-associated bacterial infection of honey bee larvae. This transcriptional pattern supports the idea of an impairment of larval development as one of the key hallmarks of EFB disease. Although, we are aware of the potential involvement of toxins in EFB disease produced by several strains, we are confident that the illustration of infection we present here is the general underlying mechanism irrespective of toxin release by the pathogen. Late-stage infected larvae showed an increase of few canonical immune genes specifically antimicrobial peptides/lysozymes and haemocytic phagocytosis-associated genes. In contrast, in an earlier stage of infection no differential transcriptional immune effector response could be detected but rather an up-regulation of complement-like and melanization cascade gene expression. However, our results do not exclude potential immune reactivity at early stages of infection. Whether these changes and their timing are due to an adaptive defence mechanism, a manipulation or damage by the pathogen, or a side effect of either both, should be an aim for future experimental work. Several putatively regulatory non-coding RNAs may act as a part of the immune effector repertoire, signaling in infected individuals or be a result of physiological change and regulation but this requires further investigations. Further, we showed an up-regulation of specific immune effectors and cellular defence markers in preparation to metamorphosis, which was not shown before in the honey bee. An interference of infection with consequential immune system activation was rarely addressed in previous studies on transcriptional response of infected honey bee larvae. Therefore, our study provides new insights into this complex relation but more experimental examinations are needed to elucidate this interrelation and to isolate the involved factors.

## Methods

### Bacteria cultivation and larvae infection

*M. plutonius* strain 49.3 (wild type strain isolated in Switzerland, [8, 10] was cultivated and used for infection as described previously (OD_600_ adjusted to 0.3 with 2.4 × 10^7^ CFU/ml; approx. 6.5 × 10^3^ CFU per larva) [7]. Freshly hatched larvae were infected with *M. plutonius* and controls received sterile medium. On day 3 post infection a subset of larvae received a diet mixed with a secondary invader (*P. alvei*, [1]) or sterile medium (19:1). *Paenibacillus alvei* (LMG 13253) was provided by BCCM/LMG Bacteria Collection (Ghent University, Ghent, Belgium) and grown in specific medium as described in [59]. In a 12 day course of in vitro rearing, infected (only *M. plutonius* and *M. plutonius* + *P. alvei*) larvae had a significantly lower survival and larval weight compared to control groups (for more details see [7]).

### Nucleic acid extraction and sample assessment

In total 3 to 5 replicate runs (24 larvae/replicate) per treatment were conducted. Samples were collected on day 3 post infection (3 dpi; *n* = 6 controls, *n* = 6 infected) and day 6 post infection (6 dpi; *n* = 6 controls, *n* = 6 infected, *n* = 6 secondary infected), respectively (Table S 1), weighed, sacrificed in liquid nitrogen and stored at − 80 °C until further processing. Total RNA and DNA were successively isolated from individual larvae. Sampled individuals were homogenized in 300 μl (3 dpi) and 600 μl (6 dpi) extraction buffer (NucleoSpin RNA purification kit, Macherey-Nagel, Düren, Germany), respectively. 50 μl (3 dpi) and 100 μl (6 dpi) of the homogenate was used for DNA extraction with the DNeasy Blood & Tissue Kit (Qiagen, Hilden, Germany). The remaining homogenate was used for RNA isolation using a NucleoSpin RNA purification kit (Macherey-Nagel, Düren, Germany) according to manufacturer’s protocol.

The pathogen load was estimated from isolated DNA samples with *M. plutonius* specific primers (*sod A* gene, forward: 5′-CAGCTAGTCGGTTTGGTTCC-3′; reverse: 5′-TTGGCTGTAGATAGAATTGACAAT-3′) relative to a honey bee reference gene (*amCOI,* forward: 5′-CCCCAGGATCATGAATTAGCAATGA-3′; reverse: 5′-TTCGGGGGAATGCTATATCAGGT-3′) via qPCR like described in [7], using *M. plutonius* genomic DNA as positive control as well as respective negative controls. Further, we determined pathogen loads as plasmid copy equivalents relative to a plasmid standard (provided by Eva Forsgren, SLU, Sweden) and took larval weight into account. Finally, we selected 4 larvae per treatment and time point for RNA sequencing, preferably with the highest RNA purity, determined on a NanoDrop 1000, and a similar pathogen load in the infected groups (or no detectable *M. plutonius* load for controls) (for further details on individual samples see Table S 9, Additional file 11).

### RNA-sequencing

The integrity of isolated RNA was verified (with the respective RIN) on an Agilent Bioanalyzer 2100 using an Agilent RNA 6000 Nano Kit as recommended by the manufacturer (Agilent Technologies, Waldbronn, Germany). The remaining genomic DNA was digested with TURBO DNase (Invitrogen, ThermoFischer Scientific, Paisley, United Kingdom). The amount of bacterial-derived rRNA sequences was reduced with the Ribo-Zero magnetic kit (Epicentre Biotechnologies, Madison, WI, USA) and the removal of eukaryotic-derived rRNA sequences was performed with the NEBNext Poly(A) mRNA Magnetic Isolation Module (New England BioLabs, Frankfurt am Main, Germany). For sequencing, cDNA libraries were constructed with a NEBNext Ultra II RNA Library Prep Kit for Illumina (New England BioLabs, Frankfurt am Main, Germany). Library sizes and quality of samples were assessed on an Agilent Bioanalyzer 2100 using an Agilent High Sensitivity DNA Kit as recommended by the manufacturer (Agilent Technologies, Waldbronn, Germany). Determination of the concentration of libraries was performed on the Qubit® dsDNA HS Assay Kit as recommended by the manufacturer (Life Technologies GmbH, Darmstadt, Germany). Sequencing was performed on a HiSeq2500 instrument (Illumina Inc., San Diego, CA, USA) using the HiSeq Rapid PE Cluster Kit v2 for cluster generation and the HiSeq Raid SBS Kit (300 cycles) for sequencing in the paired-end mode (read length of 150 bp) and running 2 × 150 cycles.

### Bacterial community analysis

Bacterial 16S rRNA gene amplicons were generated using fusion primers TCGTCGGCAGCGTCAGATGTGTATAAGAGACAGCCTACGGGNGGCWGCAG (MiSeq_B_V3_for_klindworth) and GTCTCGTGGGCTCGGAGATGTGTATAAGAGACAGGACTACHVGGGTATCTAATCC (MiSeq_B_V4_rev_klindworth) including bacteria targeting primers from Klindworth et al. [60]. The PCR reaction mixture with a total volume 50 μl contained 1 U Phusion high fidelity DNA polymerase (Biozym Scientific, Oldendorf, Germany), 5% DMSO, 0.2 mM of each primer, 200 μM dNTPs, 0.2 μl of 50 mM MgCl_2_, and 25 ng of isolated DNA. Thermal cycling scheme for bacterial amplicons was as follows: initial denaturation for 1 min at 98 °C, 25 cycles at 98 °C for 45 s, 45 s at 60 °C, and 30 s at 72 °C, and a final extension at 72 °C for 5 min. The resulting PCR products were checked by agarose gel electrophoresis for appropriate size and purified using the MagSi-NGS^PREP^ Plus Magnetic beads (Steinbrenner Laborsysteme GmbH, Wiesenbach, Germany) as recommended by the manufacturer. PCR products were used to attach indices and Illumina sequencing adapters using the Nextera XT Index kit (Illumina, San Diego). Index PCR was performed using 5 μl of template PCR product, 2.5 μl of each index primer, 12.5 μl of 2× KAPA HiFi HotStart ReadyMix and 2.5 μl PCR grade water. Thermal cycling scheme was as follows: 95 °C for 3 min, 8 cycles of 30 s at 95 °C, 30 s at 55 °C and 30 s at 72 °C and a final extension at 72 °C for 5 min. Quantification of the products was performed using the Quant-iT dsDNA HS assay kit and a Qubit fluorometer (Invitrogen GmbH, Karlsruhe, Germany) following the manufacturer’s instructions. MagSi-NGS^PREP^ Plus Magnetic beads (Steinbrenner Laborsysteme GmbH, Wiesenbach, Germany) were used for purification of the indexed products as recommended by the manufacturer and normalization was performed using the Janus Automated Workstation from Perkin Elmer (Perkin Elmer, Waltham Massachusetts, USA). Sequencing was conducted using the Illumina MiSeq platform with dual indexing and MiSeq reagent kit v3 (600 cycles) as recommended by the manufacturer (Illumina). CASAVA software (Illumina) was used for demultiplexing and clipping of adapter sequences from the raw amplicon sequences. Fastp (ver. 0.19.6) [61] with a minimum phred score of 20, a minimum length of 50 base pairs, a sliding window size of four bases, was used for read correction by overlap and adapter removal of the Illumina Nextera primers. Paired-end reverse reads were merged with the paired end read merger (PEAR ver. 0.9.11) [62] with default settings. Additionally, reverse and forward primer sequences were removed with cutadapt (ver. 1.18) [63] with default settings. Sequences were then size filtered (≤300 bp were removed) and dereplicated by vsearch (ver. 2.11.) [64]. Denoising was performed with the UNOISE3 module of vsearch and a set minimum size of 8 reads. Chimeric sequences were excluded with the UCHIME module of vsearch. This included de novo chimera and reference-based chimera removal against the SILVA SSU 132 NR database [65, 66]. Taxonomy assignments were performed with BLASTn (ver. 2.9.0+) [67] against the SILVA SSU 138 NR database with an identity threshold of 90%. Abundance bar charts were created from amplicon sequence variants with the ggplot2 package with R (R core team, 2017) and RStudio® [68].

The operational taxonomic unit (OTU) analysis confirmed that more than 99% of bacteria in the infected individuals were represented by *M. plutonius* reads compared to the control larvae (free of *M. plutonius*) in both 3 days and 6 days post infection individuals. We determined the relative compositions of the larval microbiota, which was composed of typical honey bee specific (e.g. *Fructobacillus, Lactobacillus, Bombella*, etc.) and also environmental bacterial genera (Fig. S 5, Additional file 1).

### Transcriptome analysis

We performed our analysis with trimmed paired-end raw reads. Mapping and transcript abundance quantification was carried out with Salmon (ver. 2.1.0) [69] employing the most recent available *Apis mellifera* reference genome (Amel_HAv3.1, Annotation release 104). We recovered a high read number (total fragments: 14.89 × 10^6^ ± 9.0%) and high mapping efficiency (mapping rate range: 77.3–84.6%), which was consistent overall samples (Table S 9, Additional file 11) and complies with common quality requirements. The analysis of the quantified transcripts was followed up with the ThreeDRNAseq R package (ver. 2.0.1) [70]. Read counts and transcript per million reads (TPM) were generated using tximport R package version 1.10.0 and lengthScaledTPM method [71] with inputs of transcript quantifications from Salmon. Overall, we found 27,774 expressed transcripts of 13,829 genes. Low expressed transcripts and genes were filtered based on analysis of the data mean-variance trend (count per million reads (CPM) cut-off ≥1 and min. Sample cut-off ≥4; 4 samples resembling one group, so the chance that a very low expressed transcript is most likely to be significant if expressed in the same group with low variation) yielding 17,751 transcripts of 10,394 genes (Fig. S 6 and Fig. S 7, Additional file 1). Gene and transcript read counts were normalised to log_2_-CPM using the TMM method (Fig. S 8, Additional file 1; for complete read count and TPM data see Table S 10, Additional file 13) [72]. Further, in-group consistency and between-group variation was assessed with a principal component analysis (Fig. 1) and batch-effects were corrected with RUVr function from the RUV R package (ver. 0.9.7.1) [73]. The contrast groups in Table 1 were used to compare the expression changes between conditions of experimental design. For DEGs, the log_2_ fold change of transcript abundance were calculated based on contrast groups and significance of expression changes were determined using *t*-test. *P*-values of multiple testing were adjusted with Benjamini-Hochberg method to correct false discovery rate (FDR). A DEG was significant in a contrast group if it had adjusted *p*-value < 0.05 and log_2_ FC > 1 (for full account see Table S 11, Additional file 13).

The functional annotation clustering analysis was performed with DAVID 6.8 [74]. DEGs were converted to the respective DAVID data base accessions using the gene accession conversion tool. GO term grouping (molecular function, biological process, cellular compartment), the KEGG biological pathways as well as UP Keywords and InterPro categories were included in clustering and the threshold of significant terms was set to *p* < 0.1 (for further settings see Table S 2, Additional file 4). The results of the FAC were processed and visualized with the EnrichmentMap (ver. 2.0) plugin for Cytoscape (ver. 3.8) to illustrate and contrast the cluster and term associations of the compared groups. Functional terms analysis of secondary infection (Cont vs. Inf and Cont vs. secInf) was carried out with sets of input DEGs under relaxed conditions (FDR < 0.05; − 0.585 < log_2_FC > 0.585; Table S 12, Additional file 14), up- and down-regulated genes separated, to increase resolution and then proceeded like described above.

### Correlation analysis

In *M. plutonius* infected larvae, weight is an important factor and indicator of developmental progression and infection status. We realized that although our *M. plutonius* specific primers were reliable in detecting bacterial DNA, we were not able to differentiate whether bacterial nucleic acids originated from live bacteria or accumulated from dead cells. Weight and bacterial load did not correlate well in our full set of infected individuals (Pearson *r* = 0.15, *p* = 0.62), although the subset of solely *M. plutonius* infected correlated stronger (Pearson *r* = 0.66, *p* = 0.08; Fig. S 1, Additional file 1). Hence, we did not include infection status and performed an independent correlation analysis of mRNA levels and larval weights to avoid misinterpretation of infection loads and weight changes. Moreover, this procedure enabled us to analyse infection load independent expressional changes in infected larvae. Significance thresholds of Pearson correlations of expressed gene TPMs and weight were adjusted for multiple testing (Benjamin-Hochberg: FDR < 0.05) and the functional annotation tool in the DAVID database was used to retrieve information on functions of expressed genes significantly correlated with weight with the same procedure as before (see above).

## Supplementary Information

**Additional file 1 **: **Fig. S1 - S10**. For details see Figure captions.

**Additional file 2 **: **Fig. S11**. Detailed network figure of FAC analysis clusters and term associations.

**Additional file 3 **: **Table S1**. Correlation analysis of weight and mRNA abundance.

**Additional file 4 **: **Table S2**. Detailed results of FAC analysis for temporal DEGs in control (Cont 3 dpi vs 6 dpi) and infected (Inf 3 dpi vs 6 dpi) group, respectively.

**Additional file 5 **: **Table S3**. Expressional comparison of chitin-binding/cuticula-associated protein genes.

**Additional file 6 **: **Table S4**. Expressional comparison of DUF1676 domian containing genes and the Osiris family.

**Additional file 7 **: **Table S5**. Expressional comparison of genes related to the canonical immune pathways Toll and Imd/JNK.

**Additional file 8 **: **Table S6**. Expressional comparison of candidate genes with a function in A) silk formation, storage and/or transport of energy reserves and B) related to immune response corresponding to Fig. 4.

**Additional file 9 **: **Table S7**. Expressional comparison of genes of enzymes involved in insect hormone biosynthesis.

**Additional file 10 **: **Table S8**. Expressional comparison of heme domain-containing protein genes.

**Additional file 11 **: **Table S9**. Summary of read mapping for all samples performed with mapping-based mode and a transcriptome index in Salmon.

**Additional file 12 **: **Table S10**. Read counts and TPM reads. Raw counts were generated from transcript quantifications from Salmon using tximport R package and TPMs with lengthScaledTPM method.

**Additional file 13 **: **Table S11**. List of significant DEGs by contrast group category with adjusted *p*-value < 0.05 and log2 FC > 1.

**Additional file 14 **: **Table S12**. Detailed results of FAC analysis for DEGs of 6 dpi control (Cont) versus primarily infected (Inf) and secondary infected (secInf) group, respectively.

## Data Availability

The dataset supporting the findings of this article is available in the NCBI SRA database under the BioProject accession number PRJNA745504 (http://www.ncbi.nlm.nih.gov/bioproject/745504).

## Abbreviations

20-HE: 20-Hydroxyecdyson
AFB: American foulbrood
AMP: Antimicrobial peptide
CBP: Chitin-binding protein
CPM: Counts per million
CRAL/TRIO: Cellular retinaldehyde-binding protein and triple functional protein domain
DEG: Differentially expressed genes
DPI: Days post infection
DUF1676: Domain of unknown function 1676
ECM: extracellular matrix
EFB: European foulbrood
FAC: Functional annotation clustering
FC: Fold change
FDR: False discovery rate
GO: Gene ontology
JH: Juvenile hormone
KEGG: Kyoto encyclopedia of genes and genomes
OTU: Operational taxonomic unit
PAMP: Pathogen-associated molecular pattern
PCA: Principal component analysis
PM: Peritrophic matrix
PO/ PPO: Phenoloxidase/ prophenoloxidase
PRR: Pattern recognition receptor
RIN: RNA integrity number
TPM: Transcripts per million
VG: Vitellogenin
VHDL: Very high density lipoprotein
ZP: Zona pellucida
