# Supplementary material for: In the battle of the disease: a transcriptomic analysis of European foulbrood-diseased larvae of the Western honey bee (Apis mellifera)
Source: BMC Genomics. 2022 Dec 19;23:837. doi: 10.1186/s12864-022-09075-6 (PMC9764631; doi:10.1186/s12864-022-09075-6)
Supplement: Supplementary file 1 — Additional file 1 : Fig. S1 - S10. For details see Figure captions. [file 12864_2022_9075_MOESM1_ESM.docx]

**Supplementary Figures**

**Fig. S 1**


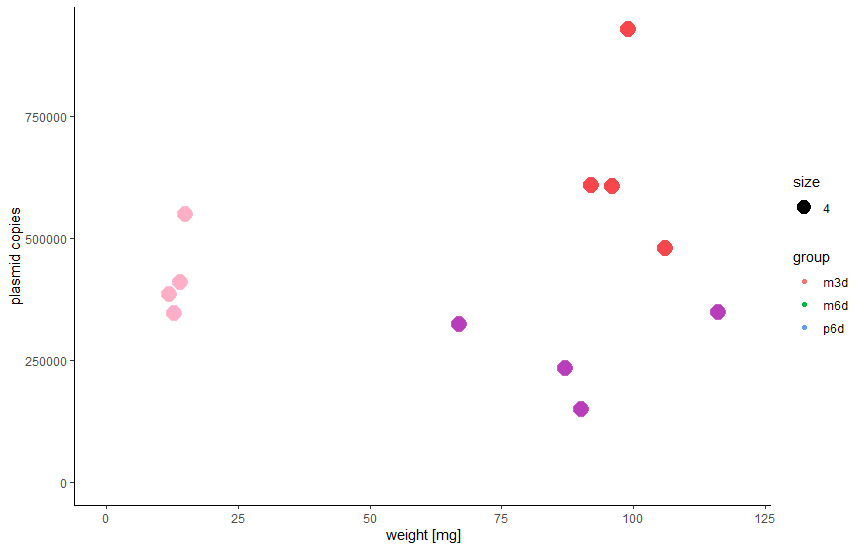


**Pearson product-moment correlation of larval weight vs. bacterial load (plasmid copy number of *M. plutonius* specific sequence; for further details see Materials and Methods).** Full data set: Pearson’s r = 0.15, p = 0.62. Subset – solely *M. plutonius* infected: Pearson’s r = 0.66, p = 0.08. Different colors represent respective groups and corresponding to Fig. 1 and Fig. S 8.

**Fig. S 2**

**
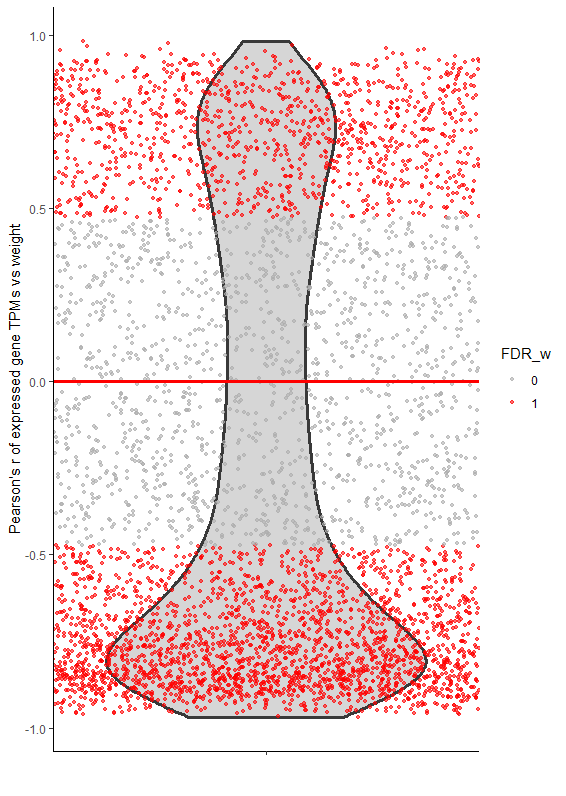
**

**Pearson correlation analysis of gene TPM vs. larval weight.** Genes significantly correlated (p < 0.05; Benjamin-Hochberg-corrected) are in red (for details see also Table S 1, Additional file 3).

**Fig. S 3**


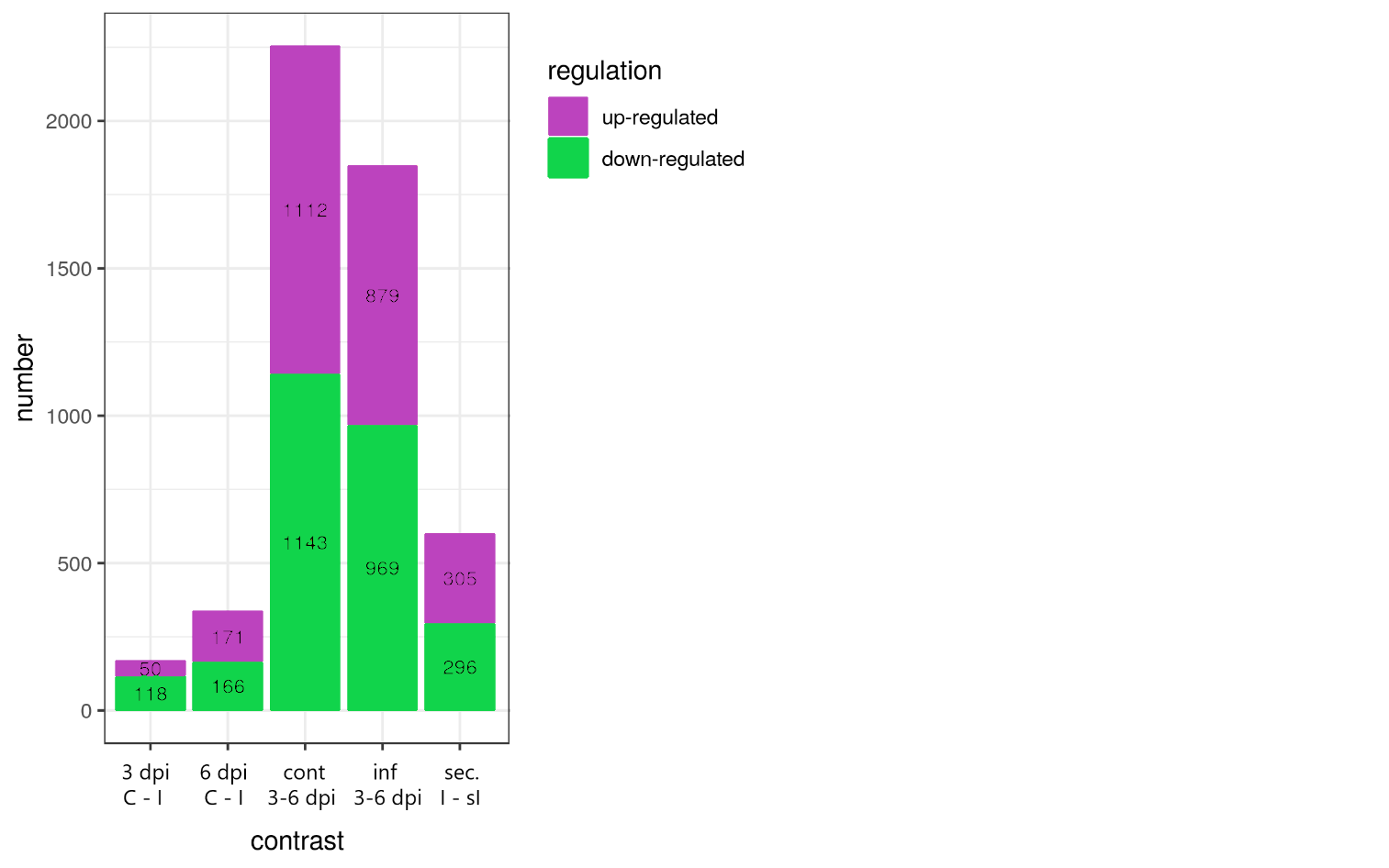


**Number of up- and down-regulated genes of considered contrasts filtered dataset.**

**Fig. S 4**


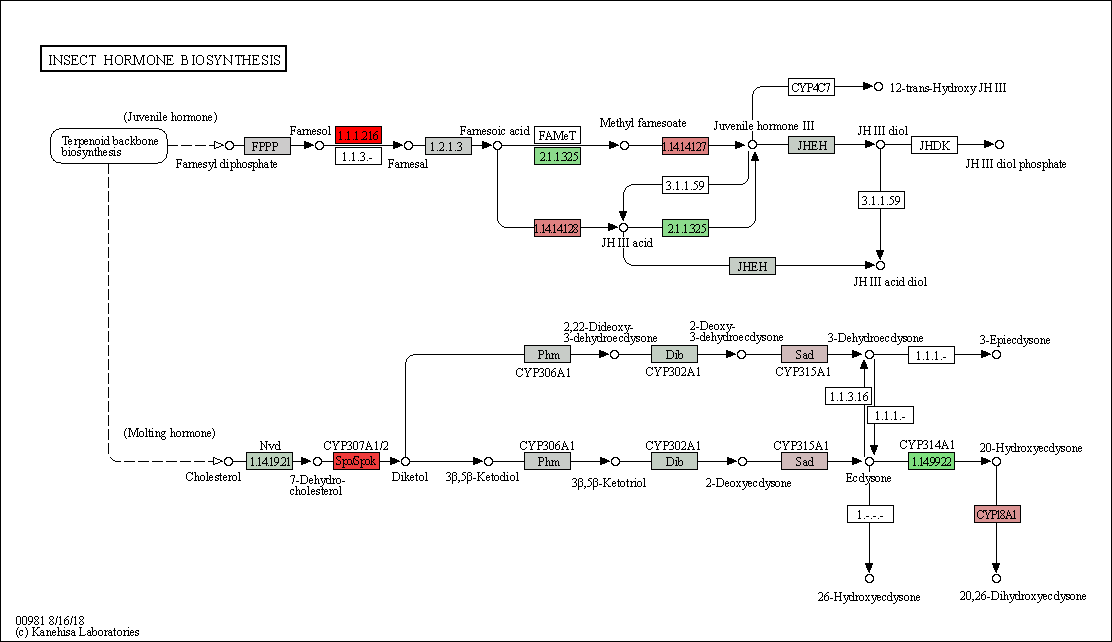


**KEGG map insect hormone biosynthesis pathway (ame00981) in 6 dpi controls vs. infected.** Red: up-regulation, green: down-regulation, grey: not regulated, white: not expressed or not annotated. For more details, see Table S 7 (Additional file 9). Created with KEGG mapper (Kanehisa et al. 2000a, b; 2023). Source (with permission from Kanehisa laboratories): https://www.genome.jp/pathway/ame00981

**Fig. S 5**


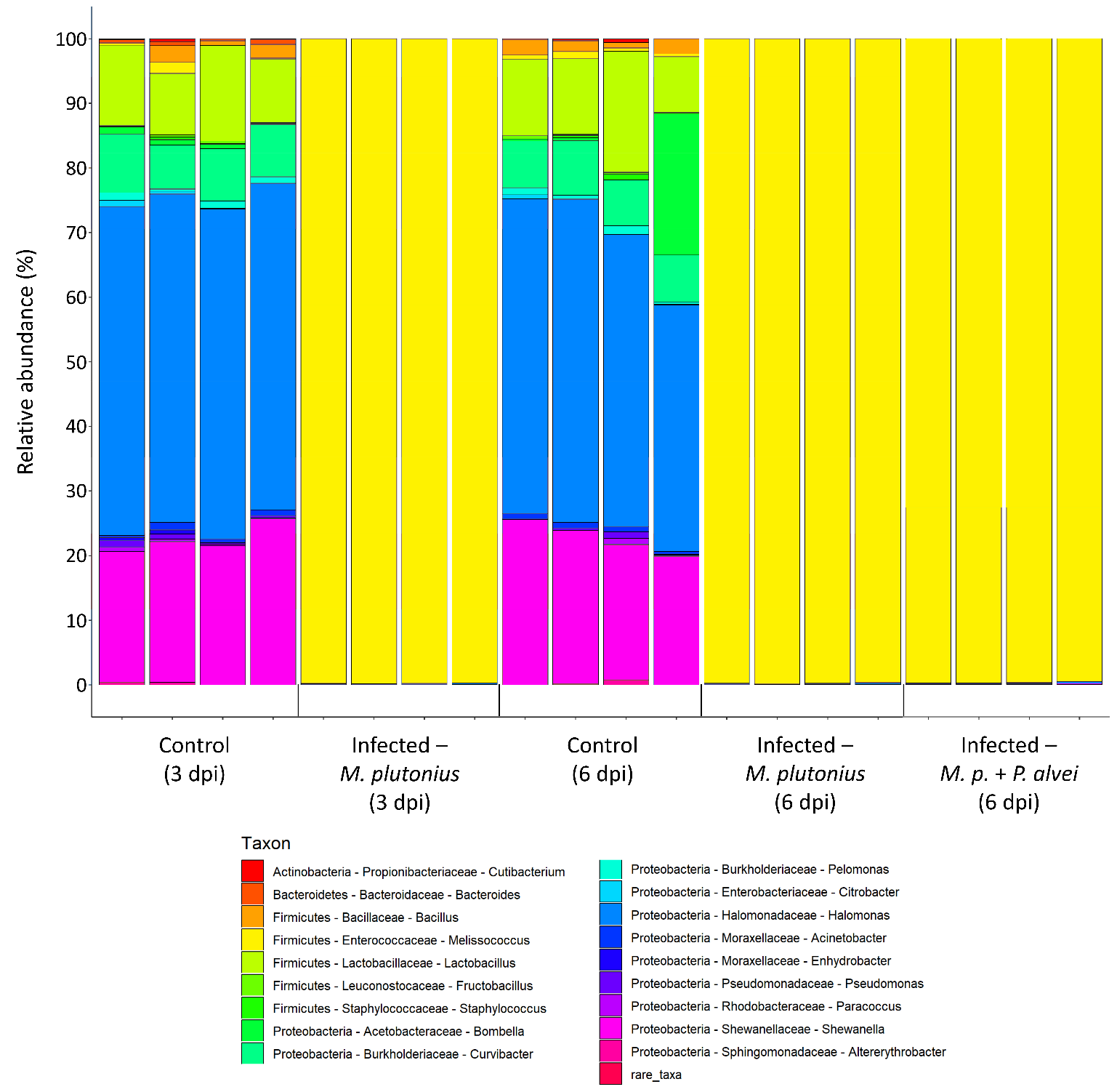


**Relative abundances of OTU composition of individual samples.**

**Fig. S 6**


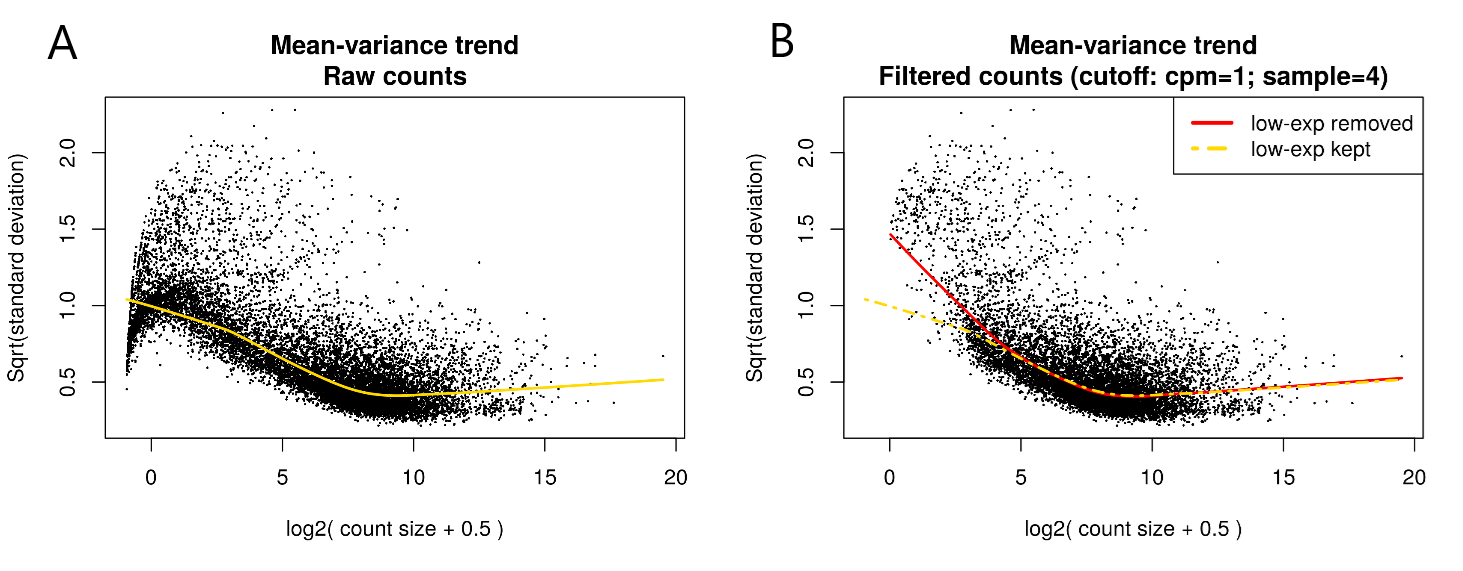
**Mean-variance trend of A raw and B filtered counts (log_2_).**

**Fig. S 7**


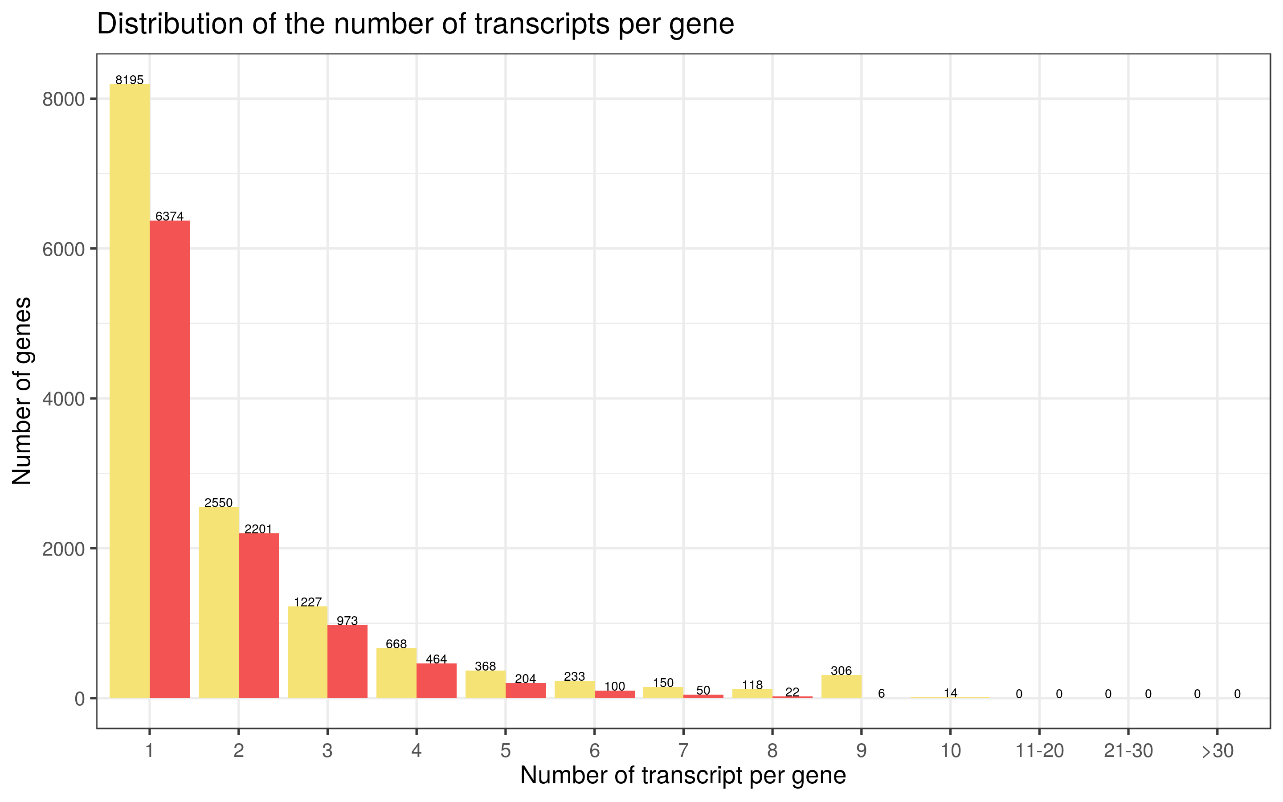


**Transcript number per gene before (yellow) and after filtering (red).**

**Fig. S 8**


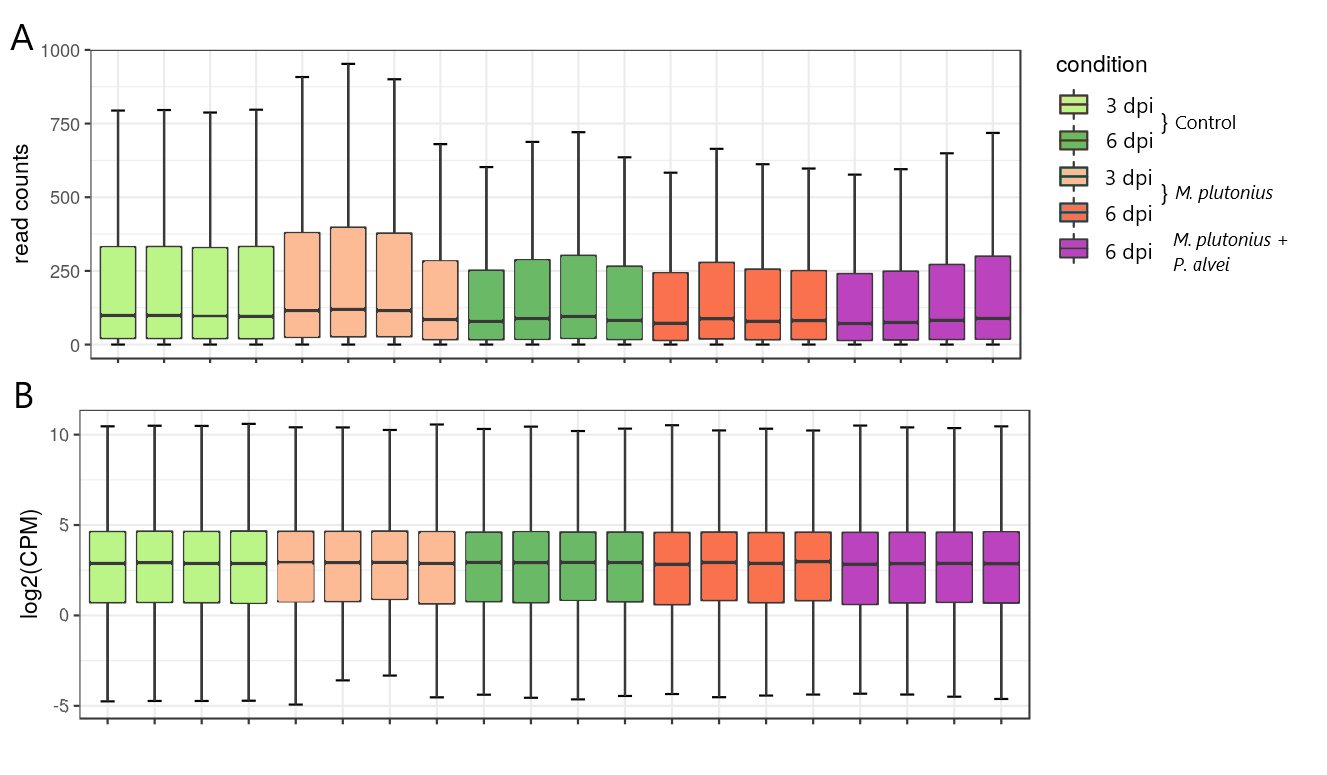


**Transcript level read counts and normalised CPM distribution across samples.** Data normalization was performed with TMM method on libraries. Different colors represent respective groups and are corresponding to Fig. 1, Fig. 2.

**Fig. S 9**


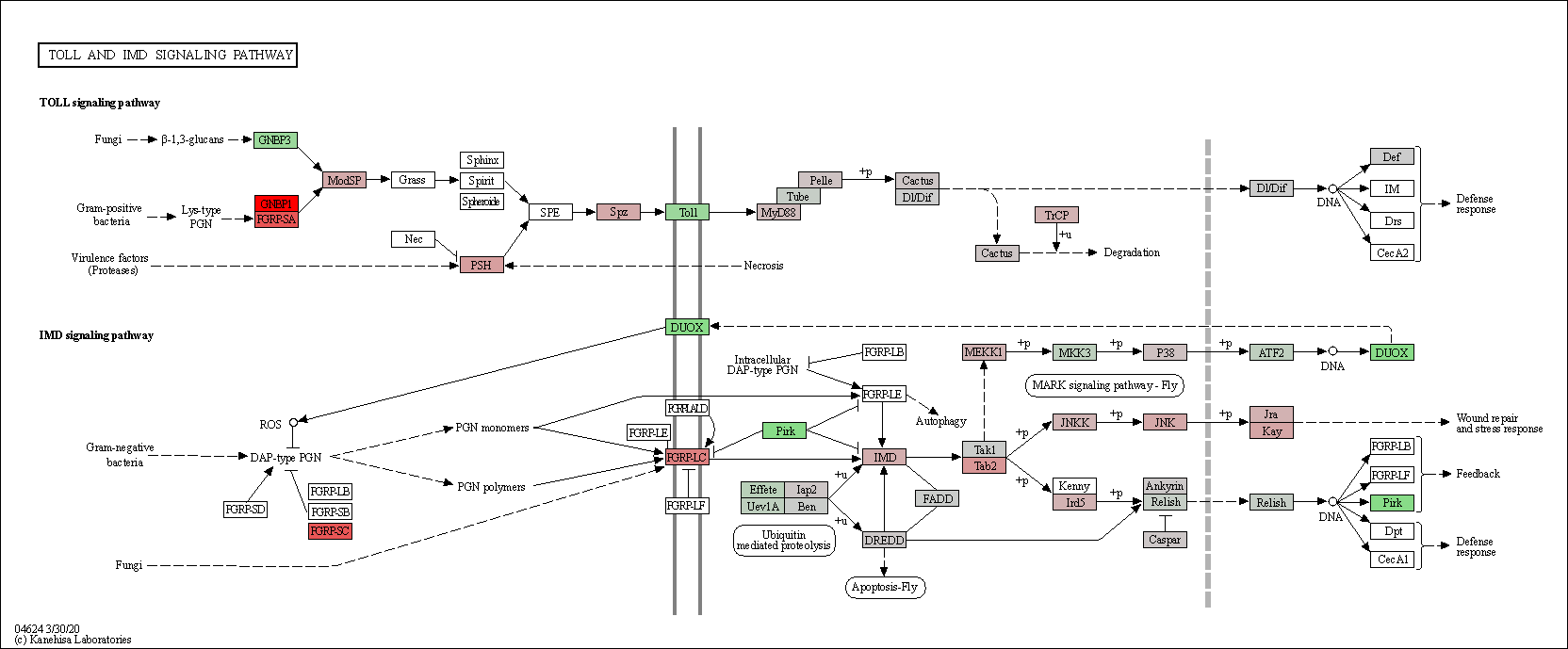


**KEGG pathway map of change in Toll-Imd signalling (ame04624) in 3 dpi vs. 6 dpi control larvae (p < 0.05).** Red: up-regulation, green: down-regulation, grey: not regulated, white: not expressed or not annotated. For more details, see Table S 5 (Additional file 7). Created with KEGG mapper (Kanehisa et al. 2000a, b; 2023)**.** Source (with permission from Kanehisa laboratories): https://www.genome.jp/pathway/ame04624

**Fig. S 10**


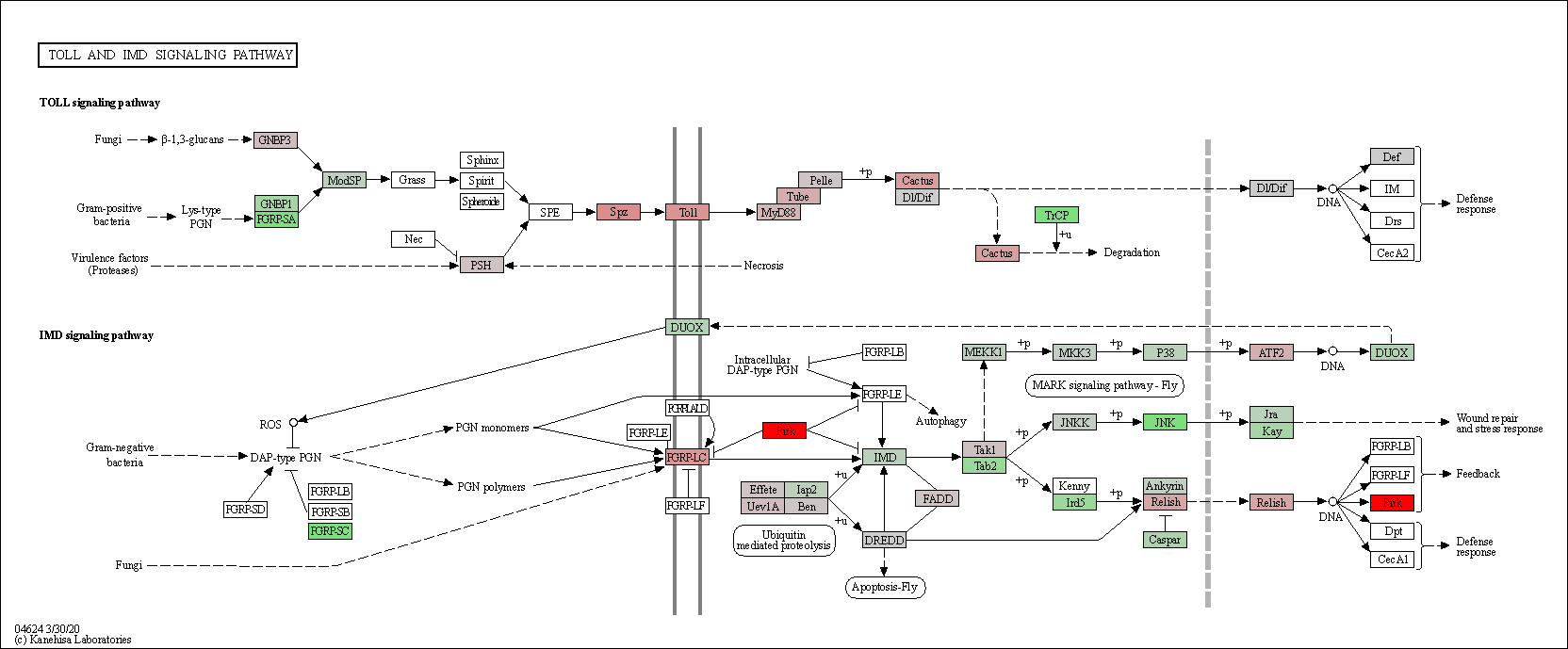


**KEGG pathway map for change of Toll-Imd signalling (ame04624) in 6 dpi infected larvae relative to 6 dpi controls (p < 0.05).** Red: up-regulation, green: down-regulation, grey: not regulated, white: not expressed or not annotated. For more details, see Table S 5 (Additional file 7). Created with KEGG mapper (Kanehisa et al. 2000a, b; 2023). Source (with permission from Kanehisa laboratories): https://www.genome.jp/pathway/ame04624

**References:**

Kanehisa M, Goto S (2000a) KEGG: Kyoto Encyclopedia of Genes and Genomes. Nucleic Acids Res. 28, 27-30. DOI: 10.1093/nar/28.1.27

Kanehisa M, Sato Y. (2000b) KEGG Mapper for inferring cellular functions from protein sequences. Protein Sci. 29, 28-35. DOI: 10.1002/pro.3711

Kanehisa M, Furumichi M, Sato Y, Kawashima M, Ishiguro-Watanabe M (2023) KEGG for taxonomy-based analysis of pathways and genomes. Nucleic Acids Res. 51 (*early online*). DOI: 10.1093/nar/gkac963
