# Supplementary material for: In the battle of the disease: a transcriptomic analysis of European foulbrood-diseased larvae of the Western honey bee (Apis mellifera)
Source: BMC Genomics. 2022 Dec 19;23:837. doi: 10.1186/s12864-022-09075-6 (PMC9764631; doi:10.1186/s12864-022-09075-6)
Supplement: Supplementary file 2 — Additional file 2 : Fig. S11. Detailed network figure of FAC analysis clusters and term associations. [file 12864_2022_9075_MOESM2_ESM.pdf]

Fig. S 11

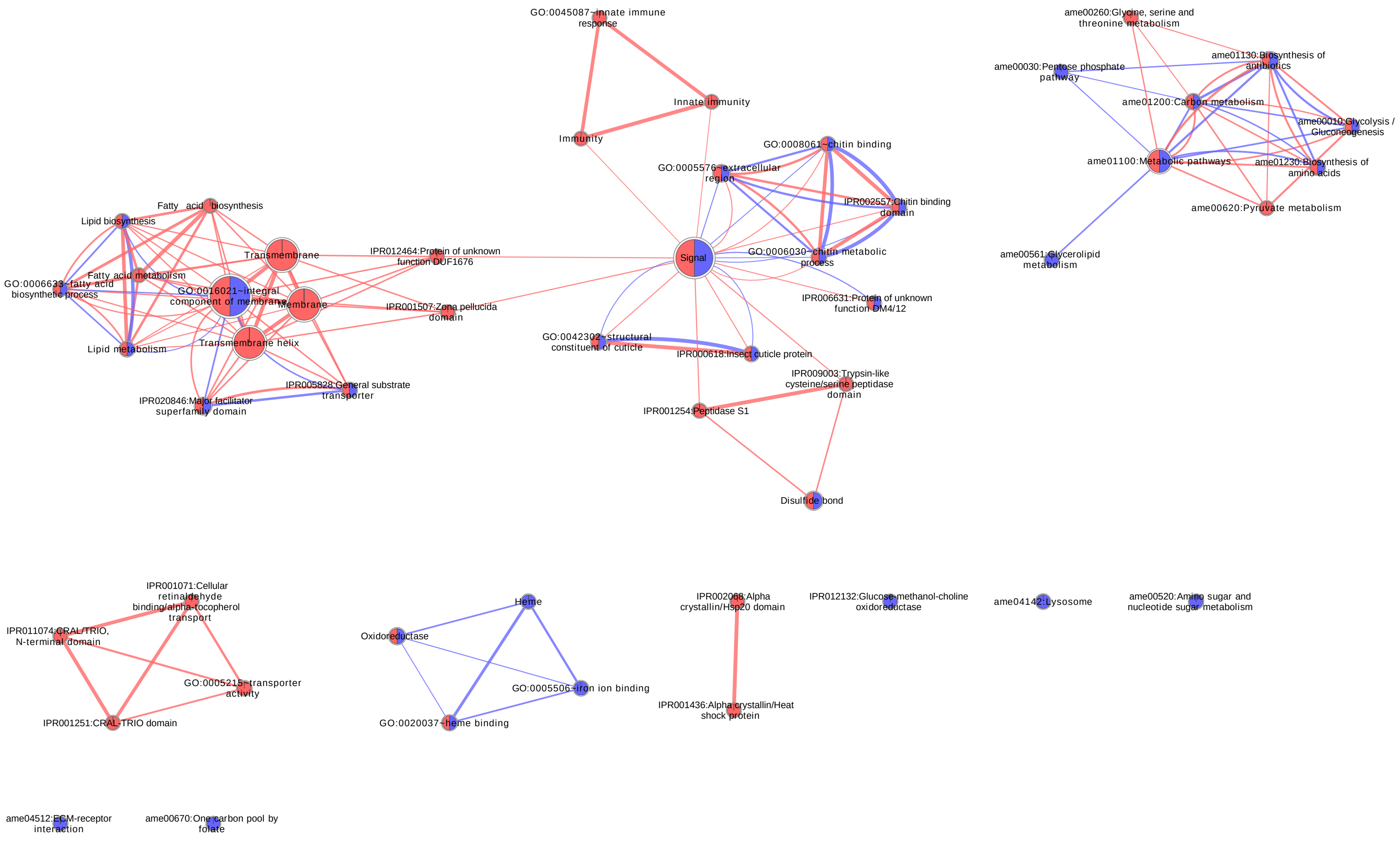

Detailed enrichment network of the DAVID functional annotation analysis. Terms are represented by circles and overlap of terms is represented by node connection resulting in clusters of terms with overlapping functions. Nodes are identified with their respective term designation. Circle size is related to enriched gene set size and edge width is positively associated with overlap of gene sets between the respective terms. For more details on underlying gene sets, numeric values and testing statistics of functional annotation clustering see Table S2 (Additional file 4).
